# “How do ethnic minority patients experience the intercultural care encounter in hospitals? a systematic review of qualitative research”

**DOI:** 10.1186/s12910-016-0163-8

**Published:** 2017-01-19

**Authors:** Liesbet Degrie, Chris Gastmans, Lieslot Mahieu, Bernadette Dierckx de Casterlé, Yvonne Denier

**Affiliations:** 1 Centre for Biomedical Ethics and Law, Department of Public Health and Primary Care, Faculty of Medicine, KU Leuven, Kapucijnenvoer 35, blok D, box 7001, Leuven, 3000 Belgium; 2 Academic Centre for Nursing and Midwifery, Department of Public Health and Primary Care, Faculty of Medicine, KU Leuven, Kapucijnenvoer 35 blok D, box 7001, Leuven, 3000 Belgium

**Keywords:** Cultural diversity, Cross-cultural, Immigrants, Minority groups, Healthcare, Systematic review, Qualitative research, Experiences

## Abstract

**Background:**

In our globalizing world, caregivers are increasingly being confronted with the challenges of providing intercultural healthcare, trying to find a dignified answer to the vulnerable situation of ethnic minority patients. Until now, international literature lacks insight in the intercultural care process as experienced by the ethnic minority patients themselves. We aim to fill this gap by analysing qualitative literature on the intercultural care encounter in the hospital setting, as experienced by ethnic minority patients.

**Methods:**

A systematic search was conducted for papers published between 2000 and 2015. Analysis and synthesis were guided by the critical interpretive synthesis approach.

**Results:**

Fifty one articles were included. Four dimensions emerged, describing the intercultural care encounter as (1) a meeting of two different cultural contexts of care, (2) in a dynamic and circular process of (3) balancing between the two cultural contexts, which is (4) influenced by mediators as concepts of being human, communication, family members and the hospital’s organizational culture.

**Conclusions:**

This review provides in-depth insight in the dynamic process of establishing intercultural care relationships in the hospital. We call for a broader perspective towards cultural sensitive care in which patients are cared for in a holistic and dignity-enhancing way.

**Electronic supplementary material:**

The online version of this article (doi:10.1186/s12910-016-0163-8) contains supplementary material, which is available to authorized users.

## Background

Worldwide, societies are becoming increasingly multi-ethnic due to the volume, speed and diversity of modern migration flows [1]. The historical presence of indigenous populations and the heterogeneity in modern migrant populations present healthcare services with a multitude of intercultural challenges. Primary causes of these challenges are differences in health determinants, needs and vulnerabilities. Despite these intercultural challenges, healthcare services should ensure culturally appropriate healthcare for every ethnic minority patient [1]. As yet, however, literature still shows disparities in healthcare, inequalities and barriers in access, lower quality of care and lower health outcomes for these patients [1–3].

Particularly challenging is the intercultural care encounter in the hospital setting because care here, is acute, necessary and inevitable during the hospitalization. The possibility of providing good intercultural care in this context is, however, challenged by language barriers, lower health literacy and higher socioeconomic stressors in ethnic minority groups, scarcity in hospital resources (time, money and people), differences in cultural traditions, differences in understanding illness and treatment and negative attitudes among patients and caregivers [2, 4–6]. Caregivers are often confronted with the intercultural reality in hospital care practices [5, 7–9] in which they try to find a dignified answer to a situation of human vulnerability [10]. Although the concepts of transcultural nursing, culturally appropriate care and cultural competence have gained a lot of interest within the literature, [11, 12] ethical guidelines on good practices regarding intercultural care are still lacking, leaving care practices open to many misunderstandings based on intercultural differences [13, 14]. Moreover, a better understanding of the bedside care experiences from the ethnic minority patients’ point of view is crucial in finding an answer to the fundamental question on how to provide good intercultural care.

Qualitative research shows increasing attention for intercultural care experiences in hospital settings although studies that provide a meaningful synthesis of these empirical findings are scarce [15]. Existing reviews on intercultural care experiences, are restricted to the caregiver’s perspective [15, 16], communication [17, 18], oncology care [16, 19] or maternity care [20–22]. Although we did not exclude these issues of interest nor settings, we aimed to gain insight in the broader bedside experience and the overall hospital context. Therefore, we aim to fill this gap by conducting a systematic review of qualitative research to explore the intercultural care experiences of ethnic minority patients admitted to the hospital.

## Methods

We carried out a review of qualitative literature based on the critical interpretive synthesis (CIS) approach [23]. Due to the large amount of data and the diversity in used methodologies we opted for an approach in analysis that is both systematic and iterative [23]. This approach is specifically intended for analysing primary qualitative research and particularly useful for generating new concepts by induction and interpretation [23].

### Search strategies

**Fig. 1:**
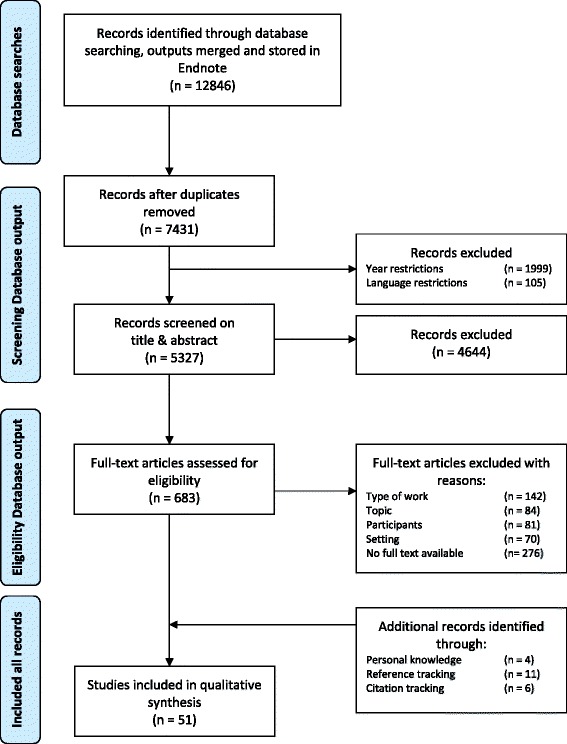
Flow chart

Four strategies were combined in sampling relevant articles [24]. First, we performed exploratory hand-searches to identify keywords and terminology relevant for building a search string. Secondly, systematic database searches were carried out in Medline/Pubmed, Embase, Cinahl and Web of Science. The same search string was used in each database, although keywords were revised when necessary (Additional file 1). Outputs were merged and stored in EndNote ×7. Duplicates were removed before screening both titles and abstracts for eligibility. Full texts of potentially relevant articles were retrieved and carefully assessed for inclusion. Thirdly, additional articles were identified based on the existing expertise and personal knowledge of the multidisciplinary research team. Each member was alert to serendipitous discoveries in his or her academic field [23–26]. Finally, we performed two rounds of citation and three rounds of reference tracking until no additional data were found [24, 26, 27]. Figure 1 outlines the entire search process guided by PRISMA [28].

### Selection criteria

The following selection criteria were used throughout the entire search process. Primary empirical research articles with a clear qualitative methodology, published as a journal article between January 2000 and March 2015 were included. Only articles in English, German, Dutch or French were eligible due to the author’s command of these languages. Books, book chapters, editorials, dissertations, reviews, theoretical articles, conference papers and letters were excluded. In order to be included, articles had to focus on (aspects of) the one-on-one care encounter between caregivers of ethnic majority and patients of an ethnic minority group within a hospital setting. Articles with a focus on cross-cultural comparisons were excluded. Articles involved with primary care, day care or outpatient settings were excluded because of the lack of bedside care experiences. Only articles with a focus on the perspective of adult ethnic minority patients were included. Studies with a focus on the perspectives of caregivers, community members, relatives, medical tourists, children and medical students were excluded. The lack of consensus in terminology used in the literature led us to include all patients with a refugee, asylum or migration background as well as patients belonging to an indigenous minority group. Nevertheless, we excluded perspectives of asylum-seeking refugees because their illegal status influences their healthcare experiences in a very specific way [8]. In this review, patients will be referred to by the overarching term ‘ethnic minority patients’ [16]. Whenever necessary, reference will be made to the specific patient group. Articles with a mixed-methodology, with multiple perspectives or multiple settings, were only included if the results were clearly separated. The full process was guided by regular discussions within the research team [23].

### Search outcome & quality assessment

The search process resulted in the identification of 51 relevant articles covering 47 studies. Characteristics of the included articles were summarized (Additional file 2). The following settings were described: maternity care (*n* = 29), general hospital care (*n* = 13), acute care (*n* = 2), oncology care (*n* = 2), mental healthcare (*n* = 1). The studies were conducted in Europe (*n* = 15), USA (*n* = 10), Canada (*n* = 10), Australia (*n* = 9), New Zealand (*n* = 1), Iran (*n* = 1) and South Africa (*n* = 1). As for the design, studies used interviews (*n* = 31), focus groups (*n* = 6), and combinations of interviews and/or focus groups and/or observations (*n* = 10). Only articles written in English met the inclusion criteria. The included articles were appraised on their quality by a sensitivity analysis [17, 29, 30]. This analysis took into account the rigor of each article as well as its relevance to our research question, which resulted in a relative contribution score (low, medium, high) (Additional file 3) [17, 29, 30]. Rigor was based on the clear description of: aim, background, design, sampling, data collection and analysis, ethical considerations and study results [30]. We used this sensitivity analysis in order to detect articles with a high contribution, which then served as a starting point in analysing the relevant data [26].

### Data extraction & synthesis

The included articles were read several times to obtain familiarity with the data, complete the sensitivity analysis (Additional file 3) and develop the table of characteristics (Additional file 2). Three rounds of analysis were performed and important passages were isolated, summarized and related. A grid was developed in order to reach an overarching view on the main recurring themes as well as on the higher level concepts. Within this process, emerging themes grounded in the data were constantly compared with the higher level concepts. After the first round of 16 articles with a high contribution, the main concepts were discussed in the research team until consensus was reached. After the second round, the analysis of the remaining “high” articles was completed. As such, a conceptual scheme [31] was developed in order to clarify the relation between the different concepts [23]. After discussing the conceptual scheme, the remaining articles with a medium or low contribution were analysed. In this last round, nuances were added but no new concepts were found. Consistent with CIS, a critical inquiry of the underlying notions about intercultural care experiences was an essential part of the synthesis process.

## Results

In our synthesis, we distinguish four dimensions that are essential in describing the intercultural care encounter in the hospital. The first dimension presents the intercultural care encounter as a meeting of two different cultural contexts of care. The second dimension describes the intercultural care encounters as a dynamic and circular process of which the establishment of a care relationship between caregiver and patient is an essential part. The third dimension shows that the way in which ethnic minority patients deal with this process of realizing a care relationship with caregivers, occurs throughout a process of balancing between the two different cultural contexts of care. And finally, there is the dimension of influence by mediators. The process of balancing between two cultural contexts of care is essentially influenced by four mediators, namely the presence of humanity in care, communication, the role of family members and the hospital’s organizational structure.

### A meeting of two different cultural contexts of care

When ethnic minority patients are admitted to the hospital, the cultural context of the ethnic minority patient and the cultural context of the caregiver and hospital inevitable meet. Differences between these two cultural contexts are closely intertwined with differences in the very meaning of illness, health, treatment and care. As such, the intercultural care encounter in the hospital essentially is a meeting of two different cultural contexts of care.

Ethnic minority patients describe the meaning of care in terms of how they are used to take care of each other within their own community, religious and cultural context [32–37]. Patients’ expectations, preferences, attitudes and behaviours in the current hospital stay are all influenced by the culturally determined values, beliefs, practices and traditions from the patient’s cultural context of care [33, 36–39]. In this regard, Cortis refers to ([36] p.113):

*“[…] the strong link between perceptions of caring and Islamic values of respect for the individual’s dignity and privacy, collective values of fostering community spirit and feelings of belonging, and genuineness in interactions with others.”*

As such, ethnic minority patients inevitably carry their own cultural views regarding care with them when staying in the hospital. Within this line of reasoning, it is important to recognize this cultural context of care as a dynamic rather than a static entity. For instance, changes on a social, gender or cultural level related to the acculturation process, can also lead to changes in the cultural context of care [34, 40–47]. Moreover, each patient has unique care preferences which lead to differences regarding the cultural context of care even within the same ethnic minority group [32, 34, 43, 45, 47, 48].

When ethnic minority patients describe the caregivers’ cultural context of care, they compare this context of care with their own context and refer mainly to the differences between both [35, 49–51]. Patients are aware of differences in values, beliefs, practices and traditions on several levels, such as differences in pain expression, rooming-in practices, in the appreciation of a fast recovery, etc [34, 39, 42, 43, 48, 50, 52–54]. Hospital rules, a medicalized view and the emphasis on individualism in care are also considered to be part of the caregivers’ cultural context of care [36, 55–57]. This context, in turn, determines the way in which care is given by the hospital staff and may be very different from the patient’s own cultural context of care [34–36, 39, 43, 50, 53, 57]. Furthermore, as Wikberg et al. describe, care traditions from the caregivers’ cultural context are taken for granted and might be used as a starting point for care instead of focusing on the individual care needs of ethnic minority patients [34, 39, 51].

### A dynamic and circular care process

How do ethnic minority patients deal with such a confrontation between the two different cultural contexts of care during their hospitalization? First of all, the narratives of ethnic minority patients provide evidence for describing the intercultural care encounter as a dynamic and circular process rather than as a one-off action with a unidirectional outcome. Patients, each with their own background and culture of care, actively participate with caregivers when being admitted to the hospital, assessed, treated and discharged [43, 54, 58]. Each intercultural care encounter is understood as a dynamic and ongoing relational process which might take on different forms. This dynamic process may lead to the establishment of a meaningful care relationship, a disengagement from this relationship, or to every possible outcome in between.

Some studies describe how a meaningful care relationship between patients and caregivers is established through a dynamic process of readjusting expectations, mediating about treatment, establishing trust or settling difficulties and conflicts [34, 54, 59]. An example of such a dynamic process is illustrated by Pasco et al. in the Filipino cultural context [54]. Filipino patients expect Canadian nurses to become “one of us” and nurses can only achieve this status by going through a dynamic process of testing. This process of testing by patients will lead, in the ideal situation, to the patients’ willingness to trust caregivers and to participate in the care relationship [54]. Another example of this process is shown by Inuit patients who discuss negative first impressions which changed to feelings of appreciation towards caregivers due to the fact that patients are becoming aware of their own position as a patient in the large and complex hospital setting [59].

Other studies describe how conflicting expectations, unresolved difficulties or misunderstandings, unresolved mistrust and the inability of overcoming barriers can lead to a disengagement or disconnection in the care relationship by patients and/or caregivers [35, 36, 50, 55, 57, 58, 60–64].

Most ethnic minority patients report the coexistence of meaningful as well as disconnected care relationships [33, 35, 37, 38, 41, 44–46, 52, 56, 65–73]. In fact, every relational process between an ethnic minority patient and his or her caregiver continuously has the chance of reaching reciprocal understanding as well as running the risk of intercultural misunderstanding [35, 60, 68].

### Balancing between two different cultural contexts of care

When hospitalized, ethnic minority patients balance between the two different cultural contexts of care without having to exclude one or the other [43, 61]. This process of “balancing between” ties in closely with the dynamic and relational character typical of intercultural care encounters. This will be illustrated on the basis of three sub-dimensions i.e. (1) the known and the unknown (2) the past and the present, and (3) the care expectations and the reality of the hospital care. In this regard, it is important to acknowledge the role of the caregivers’ reaction and their (mis) understanding of this “balancing between” process as experienced by ethnic minority patients. Caregivers understanding (or lack of it), plays a major role in establishing a care relationship and as such also effects the patients’ overall hospital experiences.

#### The known and the unknown

In the first sub-dimension of the process of “balancing between” we see that ethnic minority patients balance between fitting in with the unknown hospital context and preserving what is familiar to them.

When ethnic minority patients are confronted with the necessity of a hospital stay, they have to leave their familiar context behind (e.g. families, usual activities and cultural contexts of care) in order to submit themselves to an unknown and frightening environment [39, 51, 59, 68, 73]. This hospital environment remains, at least for some part, an unfamiliar environment for most ethnic minority patients regardless potential differences in, for example, the own acculturation process or the number of previous hospitalizations [34, 56]. Entering the unknown hospital and leaving behind the patients’ familiar context causes feelings of loss, of being alone or being a stranger [35, 56, 73]. As Baker puts it ([35], p.15):

“*They described leaving a familiar world to obtain necessary services from the “White man’s” world and in the “White man’s way.” Participants found the latter world difficult to comprehend and experienced a sense of being a stranger while there.”*

Some ethnic minority patients also describe feelings of fear, intimidation and disorientation due to the clinical atmosphere and the complexity of the hospital context [51, 56, 74]. Furthermore, unknown financial organization of healthcare services, unknown hospital rules, organizational structures and subtle power relations between caregivers are easily misunderstood by ethnic minority patients [34, 35, 44, 49–51, 56, 70, 73]. It is remarkable that, in spite of the unknown character of the hospital, many ethnic minority patients express a wish to fit in and to be “normal” [43, 69, 72]. At the same time, many patients try to maintain, modify or reconstruct meaningful but lost traditions in a way that is acceptable for them [41, 49, 50, 72]. These traditions are lost to them because they have to leave their own cultural context of care behind (i.e. due to their migration and/or in leaving their communities) [43, 49].

Caregivers, who are naturally familiar with the hospital context do not always succeed in assisting ethnic minority patients to navigate throughout this strange and unfamiliar context [51, 56, 66, 74] . Caregivers’ understanding of this process of “balancing between” the known and the unknown, plays a major role in how patients are able to deal with the frightening hospital context of care as well as with the losses within their own cultural context of care.

#### The past and the present: reviving memories

The second sub-dimension of “balancing between” illustrates how ethnic minority patients are coping with memories and previous knowledge and the way in which these memories revive in the present hospital stay [43, 61].

Migrant patients predominantly refer to reviving memories and previous knowledge rooted in their country of origin. Memories from previous hospitalizations in the new country are rarely discussed in the literature. Murray et al. describe how previous care experiences in the new country increase the migrant patients’ knowledge level and confidence also in the present care [56]. Moreover, only Eckhardt et al. illustrate how migrant patients expect reciprocal misunderstandings in the present due to communication problems in previous care encounters in the new country [66].

Periods of war and violence in the country of origin caused fear and traumatic memories for many migrant patients [47, 70]. Women in particular describe how these memories revive in their maternity care in the new country [47, 70]. They remember the death of beloved ones on the way to the hospital or in surgery due to a lack of transportation, hospital infrastructure and resources in the country of origin [40, 61, 70, 75]. In their own communities, migrant women share the knowledge that giving birth is a natural process which might last for hours and might be a balance between life and death [40, 42, 55, 60, 70, 71, 76]. This shared knowledge, previous experiences of natural or complicated deliveries in the country of origin, previous traumas as well as painful memories of their own circumcision might revive in present hospital care [38, 40, 47, 55, 56, 61, 69–71, 75]. Due to this history, many patients appreciate the high standard of care in the safe environment of the new country [41, 45, 47, 52, 61, 70, 77]. Nevertheless, it is also this history that leads to patients’ fear of long-term health consequences when they are unable to follow their own traditions or rushed into their labour as well as fear of dying from treatments such as a caesarean section [40, 55, 56, 61, 69, 71, 75, 76]. Here, a difference in meaning is caused by patients’ preference for a natural delivery and their fear of dying from the clinical treatment and the caregivers’ wish to prevent death by the same treatment from a medicalized point of view [55, 75]. Moreover, some patients questioned the competence of caregivers due to the differences in treatment approach and pain management in the new country compared to the country of origin [45]. The cultural meaning of female circumcision is another example in which patients have to balance between differences in meaning. In the past, they felt normal in having a circumcision and caregivers in the country of origin knew how to handle complications during the delivery [61]. In the new country, they balance between their gratitude of the high quality of care and dealing with the stigma of being circumcised as well as dealing with the caregivers’ lack of knowledge in handling complications due to this circumcision [40, 47, 52, 56, 69, 71]. Caregivers with knowledge, on the contrary, are highly appreciated [47, 69]. Female circumcision causes the chance of double shame for patients due to the fact that they feel shame in the new country by making one choice regarding circumcision and shame in the country of origin by making the opposite one [61].

A similar balance is found for Indigenous (Inuit and Aboriginal) minority patient groups. Memories of care experiences from smaller hospitals in the own communities revive in the present experiences in the larger hospital outside these communities [59]. Most patients appreciate being in the larger hospital with the availability of competent caregivers and medical technology although they have to wait much longer and have to deal with differences in the meaning of illness, treatment and care [39, 59, 73]. Aboriginal people, for instance, belief that illness and pain can be caused by breaking a tradition or by a violation of taboos in the external world [39]. Due to this stigma, patients are too ashamed to complain about illness and pain [39]. This understanding of pain as related to the external world, is in contrast with the caregivers’ perspective in which pain is caused by a malfunction of the human body [39].

However, one study illustrates a slightly different impact of the reviving memories and history for African American minority patients [64]. A history of discrimination and racism negatively influences these patients’ self-image and make them feel marginalized in the society. This feeling of being marginalized is also visible in the hospital setting. A greater need for caregivers’ reassurance is noticed by these patients [64].

In general, language difficulties and ethnic minority patients’ shame or reluctance in discussing this history as well as the unawareness and limited discussions by caregivers lead to difficulties in this “balancing between” process [38, 52, 55, 71]. Patients’ reviving memories and knowledge, their lack of knowledge regarding medical procedures, their fear about the medical treatment and their feeling that the treatment will not be effective, all might lead to the resistance or refusal of specific treatments [42, 55, 68, 75, 76].

#### Cultural expectations and the reality of hospital care

The third sub-dimension illustrates how ethnic minority patients balance between expectations and preferences from the own cultural context of care on the one hand and the reality of their experiences in the hospital context on the other hand. An essential aspect of this dimension is the way in which these expectations or preferences are handled or mediated by patients as well as their caregivers. It is important to notice that each ethnic minority patient has unique expectations and preferences with regard to care, embedded in his or her specific cultural context. Nevertheless, various themes are recurrently discussed in the literature.

Religion and praying are an intrinsic part in the daily lives for many ethnic minority patients [32, 33, 40, 41, 46, 63–65, 75, 76, 78]. Many give meaning to their illness, treatment and hospital care by means of their faith in God or a higher spiritual being [40, 65, 67, 75, 78]. In this regard, many patients expect to be able to pray, to conduct practices to preserve these beliefs or to receive spiritual guidance during their hospital stay [46, 63, 64]. Maintaining privacy, modesty and being cared for by female caregivers are preferences linked to the cultural and religious context of many ethnic minority patients [33, 34, 38, 46, 49, 52, 54, 56, 59, 67, 76]. Especially Muslim patients have a strong request for female caregivers and male caregivers are only accepted if all the other options are excluded [34, 63, 67, 76]. African migrants, on the contrary, accept male caregivers despite their preferences for female caregivers because these caregivers are part of the healthcare system in the new country [56]. Other evidence, on the contrary, shows that some ethnic minority patients find it more important to have a competent caregiver or a caregiver with shared cultural features, shared language or shared commonalities [53, 54, 65, 79].

Cultural care practices and traditions such as food traditions, hygiene requirements and the importance of patient’s rest are emphasized by many ethnic minority patients [34, 36, 41–44, 46, 70, 72, 74, 80]. For instance, African and Asian patients highly value traditional confinement practices for the mother after delivery (e.g. “sitting in the month”, "40-days") [41, 43, 46, 49, 50, 56, 61, 69, 70, 79–81]. Most Asian patients also expect to maintain the cosmological balance (ying & yang, hot & cold) and expect to continue the use of alternative remedies [43, 50, 67, 70, 79]. These practices are deemed important for the African and Asian patients’ long-term health although some of them might be in conflict with the use of analgesia or with a surgery like a caesarean section [42–44, 50, 56].

Culturally determined values and silent knowledge embedded in ethnic minority patients’ cultural context of care, also influence their expectations [35, 39, 54, 59, 70]. For instance, the informal rule of conduct: *“people should do things without being asked”,* or *“nurses just know, they see within”* influences respectively Mi’kmaq and Aboriginal patients’ care expectations [35, 39]. Avoiding shame through maintaining self-control, unassertiveness and enduring pain silently, are inherent in the Asian cultural context [43, 53, 54, 70, 80]. Also Sudanese and aboriginal patients try to endure pain silently [39, 42]. Underlying values of docility in the ethnic minority patients’ cultural context of care, however, can also lead to an unquestionable confidence in the medical expertise of caregivers [43, 65, 77].

From the patients’ point of view, we notice different ways of balancing between these culturally-based expectations and the reality of the hospital context. Some ethnic minority patients expect a similar way of caring by caregivers as known from their own cultural context of care [36, 39, 59]. Other patients are more aware of the contrast between their own cultural expectations regarding care and treatment and those of their caregivers embedded in the biomedical context [35, 42, 43, 57, 60, 68, 76, 82]. And still another group of patients do not expect caregivers to be aware of their cultural context of care [34, 72]. They describe, for instance, religion as a private matter, also towards their caregivers [34, 72]. In other examples, patients do not expect caregivers to understand or to speak their language [34, 66]. As such, ethnic minority patients differ individually in how they balance between maintaining cultural expectations and the reality in the hospital context of care.

This sub-dimension also draws attention to the supporting or discouraging role of the caregivers. Their awareness, understanding, respect or willingness to learn from the patient’s cultural context positively contribute to this process of “balancing between” [32, 35, 51, 63, 79]. Many ethnic minority patients appreciate caregivers who are sensitive to their rights of privacy, who encourage them to pray and who assist with their hygiene or diet requirements [36, 64]. These care relationships, enable patients to maintain or modify meaningful cultural or religious traditions in the reality of the hospital [36, 41, 72].

Caregivers’ unawareness, lack of knowledge, lack of respect and lack of sensitivity to the patients’ cultural and religious context can impede this process of “balancing between’ [33, 34, 36, 37, 45, 50, 52, 60, 63, 64, 70, 71, 73, 79]. Caregivers may react with frustration, anger, insults or stereotypes in answering the patients’ cultural-based expectations [52, 63, 71]. Some ethnic minority patients also describe uncaring attitudes and the lack of assistance by their caregivers due to differences between the two cultural contexts of care [41, 46, 50, 52, 67, 77]. Reciprocal misunderstandings in such relationships can inhibit patients to maintain meaningful traditions in the hospital and might lead to a lack of congruence between ethnic minority patients’ expectations and the reality of their care experiences [34, 36, 41, 49–51, 60, 63, 81].

### Mediators

From our critical synthesis of the literature, we present four crucial factors that are working as a mediator: (1) humanity in care, (2) communication, (3) the role of the family and (4) the hospital’s organizational culture. All four mediators work as a facilitator or as a barrier in realizing the balance between the different cultural contexts of care as well as in the process of establishing an intercultural care relationship.

#### Humanity in care

When ethnic minority patients illustrate good care experiences and meaningful care relationships with caregivers, they mostly refer to the presence of humanity in the attitudes of caregivers. Patients highly appreciate kind caregivers with a genuine concern for their well-being and caregivers who are flexible, attentive, empathic and respectful to their needs [32, 34–36, 56, 65, 67]. Moreover, caregivers who are willing to connect unconditionally, who are willing to share personal experiences and who show eagerness to spend time with the patients are highly appreciated [32, 34, 36, 40, 46, 54, 56].

It is remarkable that these facilitating attitudes of caregivers are centred on the caregivers’ ability to provide care for the patients as unique human beings [32]. When caregivers stress the shared humanity of people but at the same time acknowledge and accept cultural differences, patients feel valued as a human being and as a patient [35]. Ethnic minority patients discuss “equity” and “being treated as equal” as essential aspects in this regard [32, 35, 36]. Cheragi et al. illustrate this in the context of dignified care ([32] p.920):

*“The sublime essence of a human being raises the necessity of acting toward one another in a spirit of brotherhood and sisterhood; it is related to people’s equality by sharing the same humanity. The participants appreciated the healthcare staff’s high regard for the whole person and described that treating patients as equals regardless of their gender, position, race, and religion led to ensuring that they are valued as human beings.”*

Tensions in this regard are described by many ethnic minority patients when being treated differently or being encountered with racism and stereotypes [36, 45, 51, 52, 57, 63, 67, 68, 70]. In such care relationships, caregivers treat patients as a category with a static cultural context rather than as a unique human being with a very particular and dynamic cultural context [35–37, 45]. Even more, some patients expect caregivers to advocate for their needs even when this mean that they have to stand up against racism by other patients or colleagues [36].

A reluctance to provide care, lack of time, lack of flexibility and a caregiver’s focus on the technical part of care rather than on empathy contribute to tensions regarding humanity in care [33, 40, 56]. Moreover, caregivers who pretend to empathize or who are unwilling to engage on a social or emotional level put the relational process under pressure [32]. A lack of congruence between the patients’ expectations and experiences in this regard, lead to feelings of disappointment [33, 37].

Humanity in care is pictured here as a mediator in the process of balancing between the two cultural contexts of care and thus in the relational care process. In this, humanity in care can prevail and overcome cultural difficulties caused by the confrontation between the two cultural contexts. At the same time, a lack of humanity in care can also aggravate intercultural conflicts caused by this confrontation. Based on the literature, we can argue that caregivers who treat patients on grounds of a shared humanity, also show a willingness to learn and respect the patients’ cultural context of care.

#### Communication

Communication, understood as a joint responsibility, is an essential part of the relational care process although it is a complex and multidimensional phenomenon. From the literature, we distinguish five sub-dimensions in which communication acts either as a facilitator or as a source of many misunderstandings.

The first sub-dimension presents *low language ability* as the most described communication barrier for ethnic minority patients. Low language ability has an impact on the overall quality of care, access to services, the assessment of patients’ needs, the participation in the decision making process, on the medication and treatment compliance and on the patients’ satisfaction of treatment [36, 38, 56, 60–63, 65–67, 70, 72, 74, 77, 80–82].

Due to a low language ability and the shortage (or absence) of appropriate language services patients do not always succeed in understanding the caregivers, explaining their needs, expressing their preferences or asking for information [38, 56–58, 60, 62, 63, 67, 70, 80]. Some ethnic minority patients have difficulties in understanding caregivers due to the speed and complexity of the new language and the complexity in medical terminology [38, 62, 67, 68]. For others, this is even more difficult because of the absence of complex medical terms and procedures in the native language [38, 62, 68, 70]. Expressing treatment preferences and care needs are even more difficult when ethnic minority patients are too shy to speak the new language or when they are inhibited to ask questions on a deeper level due to the foreign language [74, 79].

Caregivers on the other hand, do not always succeed in understanding the patients’ needs and informing them in a comprehensible way [34, 45, 57, 61–63, 67, 70, 72, 73, 77, 79]. The lack of comprehensible information leads to a lack of understanding the diagnoses and treatment options by patients [52, 55, 62, 77, 79]. As a result, patients lack the opportunity to make an informed choice which can eventually result in a lack of controlling their own care [45, 62, 70, 73].

Difficulties in communication and reciprocal misunderstandings in this regard can inhibit ethnic minority patients and caregivers in the relational care process [34, 41, 51, 56, 78]. Many patients perceive the feeling that caregivers are not taking their health seriously because they are not listening to their needs or preferences [45, 57, 61, 62]. Patients feel upset, anxious, challenged or stressed as well as highly dependent on caregivers because of these communication problems [41, 56, 67, 70, 73]. Especially when caregivers are impatient or frustrated by the communication problems, they reinforce patients’ feelings of mistrust towards them as well as their feelings of being an inconvenience [38, 51, 57, 58, 65, 70, 74, 78]. Suurmond et al. discuss that patients might attribute the inadequacy of their care to being discriminated while it can be caused by difficulties in communication and a lack of information instead [57]. Good communication with comprehensive information, on the contrary, gives patients the opportunity to be in control of their own care and to engage in a meaningful intercultural care relationship [32, 41, 56, 67, 69, 73].

The second sub-dimension illustrates the pivotal role of *non-verbal communication*, such as body language, facial expressions, gestures, mannerisms, speech, intonation, volume, touch and gaze [36, 38, 39, 52, 54, 67, 68]. These non-verbal expressions can be very different for each cultural context. Misinterpretations in this regard, can negatively influence the intercultural care process [38, 39, 52, 54]. In some cases, ethnic minority patients feel as a study object, due to caregivers who are staring at them, pulling faces or having facial expressions of disgust towards them [36, 52, 61, 63, 68, 71]. In other cases, patients feel that caregivers are looking down on them by talking over their heads without addressing them as a person [52, 61]. In this regard, we can argue that ethnic minority patients are very sensitive to non-verbal expressions, especially when their language ability is low [54, 68].

The third sub-dimension discusses the *cultural sensitivity of communication*. In this regard, communication is interpreted by patients and caregivers according to their own specific cultural context. The crux of the matter is that ethnic minority patients and their caregivers may share the same language, but that differences due to the confrontation between the two cultural contexts may lead to a lack of shared meaning [38, 43, 68] as illustrated by Higginbottom ([38] p.300).

*“[…] Individuals may speak the same language, but due to cultural differences, such as perceptions and mannerisms including non-verbal expressions, encounters when using health care services can have different meanings for each party. A major consequence of unshared meaning seemed to be misunderstanding about what services, and response to care, they were to expect.”*

Silent knowledge and cultural values from the patients’ cultural context can cause patients to hesitate or feel embarrassed in expressing their needs, preferences as well as to express their pain and asking for care. [35, 39, 42, 53, 54, 65, 70, 73, 80] In the Mi’kmaq culture for example, patients expect caregivers to *“do things without being asked”. *[35] As such, they will hesitate to communicate their care needs or to ask information from caregivers who are, however, unfamiliar with this informal rule of conduct. [35] Other ethnic minority patients hesitate to ask for treatment or care because they do not want to be a burden for caregivers. [35, 56, 58, 65, 73] Also the cultural sensitivity of some health issues (e.g. female circumcision) enhances patients’ reluctance in discussing these health issues with caregivers. [61, 71] In our conceptualization, we notice that most of these cultural meanings are silent knowledge within the patients’ cultural context of care and are often not discussed with caregivers which might lead to a difference in meaning about what to expect from each other in the care process. Moreover, also attitudes and ethnocentric values embedded in the caregivers’ cultural context can contribute to communication difficulties [36]. In other examples, caregivers try to assist patients by using jokes or distraction techniques as known from the biomedical context [54]. Nevertheless, they fail in doing so because they start from distraction techniques which can be inappropriate in the patients’ context [54, 80].

The fourth sub-dimension illustrates the *social dimension of communication*. This is related to the concept of humanity in care as mentioned before. Many ethnic minority patients referred to situations in which caregivers are non-talkative to them, especially when it comes to personal conversations [33, 37, 51, 59, 73]. Most conversations in this regard are restricted to clinical communication about illness or treatment but are not addressing the patient as a social human being [59]. As such, caregivers who fail to see communication as a medium of integrating the patients’ social and clinical dimensions are responsible for the perceived lack of social support in care relationships [33, 37, 73]. Some patients even feel that caregivers treat them differently due to a perceived contrast in conversations between themselves and autochthone patients with the caregivers [33, 45, 51]. On the contrary, patients feel respected as a person when caregivers try to communicate with them despite communication difficulties [62]. In these cases, patients feel that caregivers take their health and care seriously [62].

The fifth sub-dimension refers to the *structural conditions* of communication. Busyness of caregivers and their lack of time puts pressure on the intercultural dialogue [72, 78]. The availability of language services or formal interpreters can improve the intercultural dialogue [66, 67] although many patients express doubts about the correct translation, the confidentiality and trustworthiness of these formal interpreters [38, 45, 46, 65, 82]. The shortage or absence of appropriate language services, especially in daily care moments, contributes to the patients’ feelings that caregivers are not motivated to facilitate communication or to engage in a meaningful dialogue [36, 58, 61, 70, 72]. Some patients as well as some caregivers consider these communication difficulties as a patient’s responsibility instead of seeing it as a joint responsibility [36, 57].

#### The role of family members as informal care providers

Another pivotal mediator is the support of family or community members as informal care providers in the hospital [50, 53, 59, 63, 69, 73]. Patients rely on the extended family members in their attempt to balance between the two cultural contexts of care and in the establishment of a care relationship with the caregivers.

Visiting the sick is an important responsibility in the cultural and religious context of many communities [35, 46, 53, 56, 67, 68]. Family members take care for ethnic minority patients in accordance with their shared cultural context by providing social support like setting them at ease and alleviate boredom, stress or anxiety during treatments or long waiting times in the hospital [35, 41, 54, 59, 68, 70, 79]. Also providing patients with proper food and praying for and with them is part of this support [41, 42, 54, 56, 70, 79]. Some family members also assist with more intimate needs such as personal hygiene [53, 54], especially when there is a perceived lack of caregivers' assistance regarding these needs [51].

Family members are deemed important in achieving proper ways of dialogue between ethnic minority patients and caregivers [41, 51, 54, 56, 59, 65–67, 72, 79]. Due to their role as preferred language facilitators or informal interpreters, they feel responsible for communicating and advocating for the patient’s needs [67]. They also feel responsible for understanding and (re) constructing illness and treatment on behalf of the patient [67]. Family members might play a major role in the decision-making process [35, 68, 78]. For some patients, treatment decisions influence the entire family which emphases even more the importance of making these decisions together with the family [68].

Ethnic minority patients rely on family members in maintaining the own cultural context in the hospital [41, 51] as well as in mediating between their own cultural context of care and that of the caregivers [35, 41, 54, 67]. This crucial role is not self-evident because it is not always easily accepted in the hospital context. For instance, limited visiting regulations and the expectation of dyad care relationships in the hospital can put pressure on the role of family members [35, 52, 68, 70, 72]. Even for patients themselves, this role is not always self-evident. Ethnic minority patients describe various difficulties due to the interpreting actions by family members. Possible examples are the patients’ embarrassment in telling family members the necessary information, family members’ failure to translate the medical terms correctly, or difficulties in translating bad news [56, 57, 62, 65, 67, 73]. Some patients are also confused when the advice from family members differs from that of the caregivers [41, 50, 74, 76]. Still others ask caregivers to act as a liaison to reduce the amount of family visitors, especially when they have to discuss sensitive care issues with their caregivers [46].

As mentioned before, ethnic minority patients have to deal with losses in the familiar context (due to the migration process and/or in leaving their communities) in order to receive hospital care [49, 70]. In this regard, patients frequently mentioned a negative impact on their well-being and recovery due to this loss of support by family members [41, 46, 49, 54, 56, 59, 66, 70, 73, 79, 80]. Consequently, many patients felt alone and isolated in the hospital [40, 70, 73, 80]. In some cases, this loss is compensated by other community members or even by caregivers [41, 46, 51, 54, 59, 63, 69, 79]. In other cases, patients might leave the hospital as soon as possible to be reunited with their families [70].

#### The hospital’s organizational culture

The organizational culture of the hospital is an essential part of the caregivers’ cultural context of care. The hospital’s organizational culture with its own regulations and implicit values highly influences the manner in which ethnic minority patients are able to “balance between”. The easy and equal access of care, the high quality of care, the availability of specialized caregivers and high medical technology provide many patients with a sense of security in the hospital [34, 41, 43, 49, 52, 59, 67, 69, 71, 72, 79, 81].

At the same time, many patients emphasize difficulties in the care process due to the hospital’s organization, such as a lack of caregivers, interpreters, bilingual staff or religious support [38, 57–59, 66, 67, 70, 74, 77]. Not only the lack of interpreters, but also their lack of time when they are available [82] or the fact that they are automatically present, [45] reduces the patients’ participation and choice in their own care. Long waiting times and the perceived busyness of caregivers impede the intercultural care relationship [33, 37, 51, 54, 56, 59, 60, 67, 74, 77].

Moreover, medical technology and the security of the hospital context are ambivalent for several reasons. For some patients, medical technology provides security on the one hand but the caregivers’ faith in this technology might contradict with the patients’ faith in religion as well [73]. For others, this technology provides a sense of security but at the same time it diminishes the control of their own body [43]. Still others prefer being in their own communities rather than being in the hospital despite its sophisticated services [70].

The hospital’s organizational culture also includes the way in which consistency of care and the continuity of caregivers are provided in the hospital [34, 54, 56, 79]. In order to establish a meaningful care relationship, it is a necessary for many ethnic minority patients to meet with the same caregivers throughout the entire hospital stay without having to repeat their needs over and over again and without having to start all over in the intercultural care process [56, 76, 79].

### Meaningful versus disconnected care relationships

Many ethnic minority patients discuss two opposite outcomes of this process of “balancing between”, namely meaningful versus disconnected care relationships. As mentioned before, both outcomes can be present and can be dynamically changed during the hospital stay [34, 59]. Baker and Daigle even prove that during the patients’ hospital stay, meaningful care relationships with reciprocal understanding can prevail over the disconnected care encounters, which are marked by misunderstandings [35].

Meaningful care relationships are the result of patients and caregivers who are able to cross the divide between the two cultural contexts of care [35]. In these relationships, patients are able to find a good balance in all its dimensions and caregivers are able to understand this process of “balancing between” and to respect the patients’ cultural context [59]. Caregivers with competence and knowledge, with a willingness to care for patients as human beings, with kindness and friendliness and who are willing to communicate on a social level and to accept the role of family support, contribute to the intercultural care process [35, 36, 69]. Meaningful relationships are described by patients as trustful relationships and being able to trust caregivers reduces the patients’ stress and it can make it easier for patients to seek for care and to follow the caregivers’ advice even when this advice is in contrast with their own cultural context of care [54, 58, 78]. One study notice that patients also have a wish to maintain a meaningful relationship because caregivers are in control of their health [78].

Disconnected care relationships, on the contrary, are the result of patients and caregivers who are unable to cross the divide between the two cultural contexts of care [35]. These relationships are the result of reciprocal misunderstandings and the patients’ inability to find a good balance, as well as the caregivers’ lack of sensitivity to this process. Caregivers with an unwillingness to care for patients as human beings, with a focus on tasks and an unwilling attitude to resolve communication problems or to accept the role of the family contribute to patients’ disengagement in the intercultural care relationship. Moreover, many patients mention caregivers with an unfamiliarity towards their needs [33–35, 37, 46, 53, 57, 61, 63, 67, 72]. Cortis et al. describe the assessment phase in the hospital as an important opportunity to get to know the individual patient though many caregivers carry out this assessment as a routine task rather than grasping the opportunity to start a meaningful care relationship [33, 36, 37].

Disconnected care relationships cause many patients to distrust, alienate or withdraw themselves and they are often a reason for patients to reject diagnoses or treatments, to leave the hospital early or to express the intention of not returning back to the hospital [35, 43, 50–52, 56, 58, 61, 74, 75]. A lack of choice and control, feelings of powerlessness and the loss of self-agency compromise the well-being of many ethnic minority patients [44, 51, 52, 56, 60, 67, 70, 77, 81]. The necessity of the patients’ hospitalization, the lack of information and the limited discussion between caregivers and patients aggravate these feelings of powerlessness and the lack of choice and control [44, 67, 68, 77, 81, 82]. As such, a well informed decision is not easy to make by these patients. This might be aggravated when family support is lost to them although some patients notice that including their family members in the decision making process diminish their own control [67, 68, 78]. Killoran et al., furthermore, illustrate how caregivers give patients a treatment choice but as a result, patients question the competence and knowledge of these caregivers [82]. From their cultural context of care, these patients expect caregivers to recommend only the best treatment option [82].

Furthermore, many patients feel vulnerable and different, alone, embarrassed and lessened as a person when having to stay in the hospital [35, 56, 61, 63, 71, 74, 81]. Some patients even blame themselves for their inability to understand the language of the new country or blame themselves for disconnected care relationships [34, 57, 67, 77]. Despite their desire to fit in and to be “normal”, patients might feel like a stranger [44, 63, 72]. Some patients feel embarrassed when they have a request for special treatment due to their cultural or religious needs [63]. Ethnic minority patients might do everything that caregivers ask even when this is against their own cultural practices [80]. Others, on the contrary, resist practices which make them feel uncomfortable although this resistance might not always be heard by caregivers [42, 52, 67, 77, 81]. Still others tolerate negative events in an apathic or passive way due to their loyalty towards their caregivers or due to fear of reprisals from caregivers when patients complain [34, 36, 39, 51, 77].

It is remarkable that many ethnic minority patients highly appreciate their care despite the negative events during the hospital stay [35, 77]. Patients’ acceptance of negative events and their hesitation to complain can be ascribed to a highly assessed cultural value on docility, subtle cultural norms, social desirability and politeness, implicit trust in the hospital and caregivers’ knowledge, previous care encounters and low expectations, lack of knowledge on how to complain, lack of knowledge on what services should provide, as well as patients’ awareness about their minority status and patriotism towards the new country [35, 62, 67, 70, 77].

In conclusion, our analysis shows that this “balancing between” process by ethnic minority patients gives them a chance of participation in both cultural contexts as well as a double chance of feelings of loss due to the differences between the two cultural contexts of care. As such, ambivalent feelings towards their hospital care experiences are present in the narratives of many ethnic minority patients.

## Discussion and conclusion

### Discussion

This is the first systematic review exploring the intercultural care experiences of ethnic minority patients in the broader hospital setting from a critical interpretive perspective. This reviews confirms the challenging character of intercultural care encounters due to language barriers, scarcity in hospital resources, differences in cultural traditions, differences in meaning of illness and treatment and negative attitudes among patients and caregivers as represented in existing literature [1, 2, 4–6]. With this review, however, it became clear that the intercultural care encounter is an even more complex interplay of various actors. The CIS, was a useful analytical lens to grasp this fundamental complexity and to envisage the intercultural care encounter as a dynamic relational process in which ethnic minority patients “balance between” their history, values, beliefs, preferences and expectations from their own cultural context of care and the actual reality in the hospital’s cultural context of care. As such, we confirm the intrinsically link between culture and care [83] but our conceptualization also discusses the dynamic process [84] and interplay between both dynamic concepts. In this, we understand the concept of culture as described by Kleinman & Benson ([85], p.1673-1674):

*“[…] culture is not a single variable, but rather comprises multiple variables, affecting all aspects of experience. Culture is inseparable from economic, political, religious, psychological and biological conditions. Culture is a process through which ordinary activities and conditions take on an emotional tone and a moral meaning for participants. […] Cultural processes frequently differ within the same ethnic or social group because of differences in age cohort, gender, political association, class, religion, ethnicity, and even personality*.”

The concept of care is understood here as a relational process of care-giving and care-receiving in which (culturally) different opinions about the human body, illness, health, good care and appropriate treatment come together [86]. As such, the intercultural care encounter can be interpreted as a dynamic process in which patients and caregivers from two different cultural backgrounds actively engage in these reciprocal relationships. This full intercultural process is influenced by four mediators; humanity in care, communication, the role of the family and the role of the hospitals’ organizational structure. The main goal in this regard is the search for meaningful care encounters in which patients feel respected and being cared for as a unique human being with a specific cultural context of care. As such, this review offers a broad and in-depth understanding of the ongoing intercultural care practices in the hospital. By doing so, we can argue that the continuous and cyclic character of the intercultural care encounter and the active participation of caregivers and patients in this process explain why it is necessary to move beyond presenting the intercultural care encounter as a one-off action with a unidirectional outcome.

How should we understand this active participation of ethnic minority patients and their caregivers in the hospital care process? How should we understand the implications of this complex interplay in realizing meaningful intercultural care encounters?

#### Ethnic minority patients’ participation as “balancing between”

First of all, our critical synthesis of the literature provides insight in the participation of ethnic minority patients in the intercultural care encounter. Ethnic minority patients admitted to the hospital are in the first place suffering due to their illness [86, 87]. In an attempt to relieve this suffering, patients (have to) participate in several care relationships while being assessed, receiving diagnoses, making treatment decisions, being treated and being discharged from the hospital [43, 54]. At the same time, patients want to preserve their dignity in spite of this suffering and increased vulnerability [86]. As with any patient, illness intervenes in various dimensions (i.e. physical, psychological, relational, social, historical and spiritual dimension) of our human existence [88]. However, in case of ethnic minority patients, this illness and suffering are embedded in their own cultural context of care, which may be very different from the cultural context of care of the hospital and caregivers. This confrontation between the two cultural contexts of care is an intrinsic part in the patients’ search process for meaningful relationships and dignified care. Finding a balance between these two cultural contexts of care is an assignment especially for ethnic minority patients with the support from all involved in the care process. Nevertheless, we did not find records in previous studies regarding this assignment for ethnic minority patients themselves. The WHO though recognizes the importance of including these patients as active players in the improvement of their health and the services they use, as well as the necessity to empower them in this regard [1]. We suggest that it is, however, also important to empower ethnic minority patients in their assignment to balance between maintaining or reconstructing meaningful traditions from their cultural context and what is possible in the cultural context of the hospital.

#### Caregivers’ participation as facilitators

Secondly, we also acknowledge the role of caregivers’ participation in this intercultural care process. Our findings confirm that this role of caregivers goes beyond offering well-known practical solutions to a fixed set of intercultural differences [85]. Similar to several transcultural nursing theories, theories of cultural competence and anthropological frameworks, evidence was clear about the assignment of caregivers’ to be culturally aware, to be cultural sensitive, to handle with cultural knowledge and cultural skills as well as to respect, to show a willingness and even a cultural curiosity to learn from the individual patient’s’ cultural context [83–85, 89]. We, furthermore, emphasize that the caregivers’ understanding of the patients’ assignment regarding the process of “balancing between” plays a major role in establishing a meaningful care relationship. Thus, it is necessary not only to shed light on the caregivers’ role but also on how they react to the ethnic minority patients' role of “balancing between” in its various dimensions. Ethnic minority patients have the difficult task to navigate in a strange environment in which their cultural traditions are at stake, they have to deal with losses in their cultural context and they have to deal with reviving memories and incongruent expectations regarding hospital care. Caregivers are assigned to help patients in this navigation between the known and the unknown, they are assigned to understand these patients’ past memories and its influence on the present care as well as to listen, to understand or to mediate patients’ expectations and their own expectations towards these patients in the hospital reality.

#### Caregivers’ response to the mediators

Thirdly, caregivers can facilitate and support the patients’ assignment by responding to the four mediators. The results regarding *humanity in care* as a mediator show that it is imperative that caregivers respect ethnic minority patients as unique human beings (with various dimensions) but at the same time also respect them as equal human beings who are suffering due to their illness [13, 86, 87]. Caregivers have to find out what is at stake for these patients [85]. They have to do this, not only in a purely clinical way by focusing on the physical suffering but also by focusing on what is at stake at the historical, spiritual, psychological and social level as embedded in the patient’s cultural context. Many difficulties regarding humanity in care still exist because the caregivers focus is very often still limited to finding a solution to physical suffering by performing technical tasks, which is typical for the Western biomedical care model [90]. Our evidence shows that the historical, psychological, social and cultural dimension of suffering are often misjudged and underestimated in intercultural hospital care. In order to overcome these difficulties, a commitment from caregivers and patients to dignity-enhancing care can be recommended [13, 86, 87]. In this dignity-enhancing care, everyone involved in the care of an ethnic minority patient has to find the most dignified answer to this situation of human suffering by finding out what is at stake (in the broad sense), by a continuous engagement in reciprocal relationships as well as by providing optimal support for the patient in all its various existential dimensions [13, 86, 87].

The role of *communication* as a mediator is also prominent in a lot of intercultural care literature. Although we do not deny the consequences of difficulties due to the dimensions of low language ability, non-verbal communication and structural conditions of communication, we did not find the same amount of attention to the social and cultural dimensions of communication in the literature. Due to the strong connection between culture and communication, [89, 91] we could ask ourselves what happens when caregivers solely focus on the low language ability of ethnic minority patients and the structural conditions in this regard. In such cases, caregivers are alarmed when they meet patients who do not speak the same language but what happens when patients do share the same language but do not share the same meaning of this language due to differences on the cultural level? [2, 89] We could argue that this part of communication is less visible than the low language ability which makes it much more difficult to resolve these tensions. Moreover, there is also less attention to the social level of communication. We learn, however, that paying attention to this relational dimension of communication is crucial because a meaningful relationship despite language difficulties can even overcome these language barriers to a large extent [38, 62].

The role of *family members as informal care providers* is presented as a pivotal mediator in the hospital context. Many ethnic minority patients highly value family members who are making treatment decisions for and with them although this contradicts with the highly placed value on privacy and individual autonomy in the clinical biomedical context [13]. Ethnic minority patients may prefer family-centred models of decision-making and may prefer social autonomy instead of the individual autonomy [13, 92]. Consequently, caregivers have to investigate the way and extent to which family members can be involved and supported in their role as mediators as well as to investigate which family members should be present when diagnoses or treatment options are discussed [68, 89].

Furthermore, within the mediator of the *hospital’s organizational culture* the results show the important task for hospitals in accomplishing continuity of caregivers and consistency in intercultural care which are imperative for the relational process. Case managers that accompany patients throughout the entire care process or even patient and family advisors in the hospital can increase the quality of care for many ethnic minority patients [93, 94].

#### Caregivers as skilled companions

A fourth dimension of understanding the active participation of ethnic minority patients and their caregivers refers to the dimension of skilled companionship in care. Our analysis shows that ethnic minority patients value mutual respect and trust as being part of meaningful care relationships. From the patients’ side, this mutual respect can imply their respect towards the caregivers’ cultural context of care which is embedded in the hospital and in the Western healthcare context [13]. Caregivers, on the other hand, can facilitate the establishment and maintenance of mutual respect and trust by focusing on two important dimensions of the care relationship, namely knowledge and skills on the one hand and companionship on the other hand [13, 86, 90, 95, 96]. These dimensions are described by Titchen in the “skilled companionship” framework [13, 90, 95, 96] which can be linked with the cultural competence model of Campinha-Bacote [84]. The first dimension as described in the “skilled companionship” framework, emphasizes the knowledge and skills through which caregivers provide the best possible care for patients from their professional expertise [13, 86, 95, 96]. Applied to the intercultural context, Campinha-Bacote also discusses the importance of cultural knowledge and cultural skill [84]. In this regard, we found some records of ethnic minority patients who express doubts regarding the medical expertise of caregivers but also records in which caregivers show a lack of cultural knowledge towards the patients’ health-related beliefs and cultural values. However, ethnic minority patients mostly refer to difficulties due to a lack of companionship. This second dimension illustrates the attentiveness and thoughtfulness in which caregivers handle as a companion towards the patients [13, 96]. As a companion, caregivers try to understand the patient’s real world from their perspective, are sensitive towards their needs and show a cultural desire to provide culturally good care [84, 90]. According with Leininger, we can argue that starting from the patients’ real world and from their perspective is of major importance in the intercultural reality [83]. Moreover, caregivers’ attitudes of attentiveness are highly important in the intercultural encounter, since the narratives of many ethnic minority patients in this review still present the caregivers’ unfamiliarity towards their needs which leaves their care requests often unnoticed [87]. Good examples of this companionship, on the contrary, are presented when caregivers are friendly and kind, when they are attentive towards patients’ special cultural requests, when they are answering their needs, when they are attentive to communication difficulties and when they are thoughtful to the role of their families. Caregivers as skilled companions also implies, especially in an intercultural context that caregivers acknowledge the uniqueness and the “otherness” of the patient in the care relationship as well as the caregivers’ self-consciousness, awareness or self-reflection about how they relate to this “otherness” [84, 86, 90, 96]. In the intercultural care relationship, it is important for caregivers to find solidarity throughout this “otherness” in order to provide dignified care.

### Conclusion

In conclusion, this review shows that participating in the intercultural care process in the hospital poses extraordinary challenges for ethnic minority patients and their caregivers. Nevertheless, despite their cultural background, these patients expect to be relieved from their suffering by a dignity-enhancing care in which they feel respected as unique human beings. The presentation of this intercultural care encounter as a dynamic and circular process in which many people are confronted with these challenges, allows us to understand why ethnic minority patients are dealing with meaningful as well as with disconnected care relationships throughout the same hospital stay.

### Strengths and Limitations

This review draws from qualitative evidence of ethnic minority patients’ hospital experiences from a wide range of countries, contexts and settings. This is both a strength and a limitation since one could argue that it is difficult to synthesise qualitative evidence with such differences in focus, methodologies, participants and settings. Difficulties in finding an equilibrium between analysing on a higher order level without losing all the nuances within our data appeared at the start of our analysis. Consequently, we intensively discussed which approach could be appropriate to help us find this balance. As such, the strength of the review was the use of the CIS-approach as a specific method that allowed us to generate higher level concepts based on themes in which data saturation was reached. The data saturation was found across the different contexts, participants and settings. Another potential limitation of this review could be our decision to eliminate studies in the hospital setting without bedside care experiences. Consequently, issues during consultations and short-time encounters between caregivers and ethnic minority patients were beyond the scope of this review. Furthermore, a differentiation in caregivers’ profession is absent in this review. Some differences were noticed between the delivery of care from nurses and doctors but were not elaborated because of the absence in data saturation in the available evidence.

Several previous reviews confirmed the findings on the importance of intercultural communication, the role of the family, the role of organizational issues, the role of caregiver attitudes and the role of cultural differences in care beliefs and practices [15, 17, 19, 21, 22, 97]. However, the strength of this review is the attempt to provide a broad and in-depth perspective in which the intercultural care encounter is described as a dynamic process of balancing between two cultural contexts of care as described in the narratives of ethnic minority patients. This argument should help caregivers and policymakers to ask new questions on how to realize good care in an intercultural context.

### Practice implications

We suggest that envisaging the intercultural care encounter as a dynamic relational process in which ethnic minority patients and their caregivers actively participate as embedded within their own cultural context of care, raise several important questions towards current clinical practices in Western healthcare systems and towards the limited focus on the technical aspects of care in the Western healthcare [90]. We discussed the underrepresentation of the patients’ historical, psychological, spiritual, cultural and social dimension in several domains of these Western healthcare practices. As such, we call for a broader clinical perspective towards cultural sensitive care in which the patient with all its dimensions is cared for from a holistic and dignity-enhancing perspective [13, 32, 86]. According to the WHO, we suggest the importance to deliberate a migrant-inclusive healthcare system that is capable to deliver healthcare in a holistic way for all patients from a patient-centred framework [1, 6].

Nevertheless, the delivery of this holistic intercultural care is being complicated due to the lack of ethical guidelines. After all, the complex search for meaningfulness in providing intercultural care and the higher vulnerability of ethnic minority patients lead to the question on how to provide good intercultural care, which is also an ethical and cultural question. Further empirical research on fundamental ethical dimensions of intercultural care is required because ethical questions on how to provide good intercultural care are never merely answered by practical solutions to cultural conflicts [13, 87]. By doing this, the concept and practice of care would be understood as a “technical art” instead of a moral practice through which caregivers and patients try to find a dignified answer to a situation of human vulnerability [86, 87]. Our review reveals that until now, empirical research has paid little attention to the fundamental ethical dimensions of the intercultural care encounter from the ethnic minority patient’s perspective.

## Additional files

Additional file 1: Full Search String. String adjustments for each database with the results (PDF 91 kb)
Additional file 2: Summary of Characteristics of the Included Articles. A summary of the characteristics of all the included articles (PDF 555 kb)
Additional file 3: Sensitivity Analysis. The results of the sensitivity analysis carried out for each included article (PDF 339 kb)

## References

[1] WHO, Health of Migrants - The way forward. Report of a global consultation. Madrid, Spain, 3–5 March 2010. http://apps.who.int/iris/handle/10665/44336. Accessed 06 June 2016.

[2] Rechel B, Mladovsky P, Ingleby D, Mackenbach JP, Mckee M (2013). Migration and health in an increasingly diverse Europe. Lancet.

[3] Scheppers E, Van Dongen E, Dekker J, Geertzen J, Dekker J (2006). Potential barriers to the use of health services among ethnic minorities: a review. Fam Pract.

[4] Priebe S, Sandhu S, Dias S, Gaddini A, Greacen T, Loannidis E, Kluge U, Krasnik A, Lamkaddem M, Lorant V, Riera RP, Sarvary A, Soares JJF, Stankunas M, Straßmayer C, Wahlbeck K, Welbel M, Bogic M (2011). Good practice in health care for migrants: views and experiences of care professionals in 16 european countries. BMC Public Health.

[5] Van Keer R-L, Deschepper R, Francke AL, Huyghens L, Bilsen J (2015). Conflicts between healthcare professionals and families of a multi-ethnic patient population during critical care: an ethnographic study. Crit Care.

[6] WHO, Europe MFH Project group 2004. http://www.mfh-eu.net. Accessed 06 June 2016.

[7] Høye S, Severinsson E (2008). Intensive care nurses’ encounters with multicultural families in Norway: an exploratory study. Intensive Crit Care Nurs.

[8] Hultsjö S, Hjelm K (2005). Immigrants in emergency care: swedish health care staff’s experiences. Int Nurs Rev.

[9] Nkulu Kalengayi FK, Hurtig AK, Ahlm C, Ahlberg BM (2012). “It is a challenge to do it the right way”: an interpretive description of caregivers’ experiences in caring for migrant patients in northern Sweden. BMC Health Serv Res.

[10] Scanlon A, Lee GA (2007). The use of the term vulnerability in acute care: why does it differ and what does it mean?. Aust J Adv Nurs.

[11] Betancourt JR, Green AR, Carrillo JE, Ananeh-Firempong O, Owusu L (2003). Defining cultural competence: a practical framework for addressing racial/ethnic disparities in health and health care. Public Health Rep.

[12] Williamson M, Harrison L (2010). Providing culturally appropriate care: a literature review. Int J Nurs Stud.

[13] Denier Y, Gastmans C (2013). Realizing good care within a context of cross-cultural diversity: an ethical guideline for healthcare organizations in Flanders, Belgium. Soc Sci Med.

[14] Devillé W, Greacen T, Bogic M, Dauvrin M, Dias S, Gaddini A, Jensen NK, Karamanidou C, Kluge U, Mertaniemi R, Riera RP I, Sárváry A, Soares JJF, Stankunas M, Marta, Straßmayr C, Welbel M, Priebe S (2011). Health care for immigrants in Europe: is there still consensus among country experts about principles of good practice? a Delphi study. BMC Public Health.

[15] Coffman MJ (2004). Cultural caring in nursing practice: a meta-synthesis of qualitative research. J Cult Divers.

[16] Van Eechoud IJ, Grypdonck M, Beeckman D, Van Lancker A, Van Hecke A, Verhaege S (2016). Oncology health workers’ views and experiences on caring for ethnic minority patients: a mixed method systematic review. Int J Nurs Stud.

[17] Paternotte E, van Dulmen S, van Der Lee N, Scherpbier AJ, Scheele F (2015). Factors influencing intercultural doctor–patient communication: a realist review. Patient Educ Couns.

[18] Schouten BC, Meeuwesen L (2006). Cultural differences in medical communication: a review of the literature. Patient Educ Couns.

[19] Elkan R, Avis M, Cox K, Wilson E, Patel S, Miller S, Deepak N, Edwards C, Staniszewska S, Kai J (2007). The reported views and experiences of cancer service users from minority ethnic groups: a critical review of the literature. Eur J Cancer Care.

[20] Balaam M-C, Akerjordet K, Lyberg A, Kaiser B, Schoening E, Fredriksen AM, Ensel A, Gouni O, Severinsson E (2013). A qualitative review of migrant women’s perceptions of their needs and experiences related to pregnancy and childbirth. J Adv Nurs.

[21] Higginbottom GMA, Hadziabdic E, Yohani S, Paton P (2014). Immigrant women’s experience of maternity services in Canada: a meta-ethnography. Midwifery.

[22] Wikberg A, Bondas T (2010). A patient perspective in research on intercultural caring in maternity care: a meta-ethnography. Int J Qual Stud Health Well-being.

[23] Dixon-Woods M, Cavers D, Agarwal S, Annandale E, Arthur A, Harvey J, Hsu R, Katbamma S, Olsen R, Smith L, Riley R, Sutton AJ (2006). Conducting a critical interpretive synthesis of the literature on access to healthcare by vulnerable groups. BMC Med Res Methodol.

[24] Greenhalgh T, Peacock R (2005). Effectiveness and efficiency of search methods in systematic reviews of complex evidence: audit of primary sources. BMJ.

[25] Greenhalgh T, Robert G, Macfarlane F, Bate P, Kyriakidou O, Peacock R (2005). Storylines of research in diffusion of innovation: a meta-narrative approach to systematic review. Soc Sci Med.

[26] Thomas J, Harden A (2008). Methods for the thematic synthesis of qualitative research in systematic reviews. BMC Med Res Methodol.

[27] Booth A (2001). Cochrane or cock-eyed? How should we conduct systematic reviews of qualitative research? proceedings of the qualitative evidence-based practice conference, taking a critical stance.

[28] Moher D, Liberati A, Tetzlaff J, Altman DG (2009). Preferred reporting items for systematic reviews and meta-analyses: the PRISMA statement. J Clin Epidemiol.

[29] Harden A (2008). Critical appraisal and qualitative research: exploring sensitivity analysis.

[30] Mahieu L, Gastmans C (2015). Older residents’ perspectives on aged sexuality in institutionalized elderly care: a systematic literature review. Int J Nurs Stud.

[31] Dierckx de Casterlé B, Gastmans C, Bryon E, Denier Y (2012). QUAGOL: a guide for qualitative data analysis. Int J Nurs Stud.

[32] Cheraghi MA, Manookian A, Nasrabadi AN (2014). Human dignity in religion-embedded cross-cultural nursing. Nurs Ethics.

[33] Cortis JD, Kendrick K (2003). Nursing ethics, caring and culture. Nurs Ethics.

[34] Wikberg A, Eriksson K, Bondas T (2012). Intercultural caring from the perspectives of immigrant New mothers. J Obstet Gynecol Neonatal Nurs.

[35] Baker C, Daigle MC (2000). Cross-cultural hospital care as experienced by Mi’kmaq clients. West J Nurs Res.

[36] Cortis JD (2000). Perceptions and experiences with nursing care: a study of Pakistani (Urdu) communities in the united kingdom. J Transcult Nurs.

[37] Cortis JD (2000). Caring as experienced by minority ethnic patients. Int Nurs Rev.

[38] Higginbottom GMA, Safipour J, Yohani S, O’brien B, Mumtaz Z, Paton P (2015). An ethnographic study of communication challenges in maternity care for immigrant women in rural Alberta. Midwifery.

[39] Fenwick C, Stevens J (2004). Post operative pain experiences of central Australian aborginal women. What do we understand?. Aust J Rural Health.

[40] Essén B, Johnsdotter S, Hovelius B, Gudmundsson S, Sjöberg N-O, Friedman J, Östergren P-O (2000). Qualitative study of pregnancy and childbirth experiences in somalian women resident in Sweden. BJOG.

[41] Grewal SK, Bhagat R, Balneaves LG (2008). Perinatal beliefs and practices of immigrant Punjabi women living in Canada. J Obstet Gynecol Neonatal Nurs.

[42] Higginbottom GM, Safipour J, Mumtaz Z, Chiu Y, Paton P, Pillay J (2013). “I have to do what I believe”: Sudanese women’s beliefs and resistance to hegemonic practices at home and during experiences of maternity care in Canada. BMC Pregnancy Childbirth.

[43] Cheung NF (2002). The cultural and social meanings of childbearing for Chinese and Scottish women in Scotland. Midwifery.

[44] Cheung NF (2002). Choice and control as experienced by Chinese and Scottish childbearing women in Scotland. Midwifery.

[45] Herrel N, Olevitch L, Dubois DK, Terry P, Thorp D, Kind E, Said A (2004). Somali refugee women speak out about their needs for care during pregnancy and delivery. J Midwifery Women’s Health.

[46] Missal B, Clark C, Kovaleva M (2015). Somali immigrant New Mothers’ childbirth experiences in Minnesota. J Transcult Nurs.

[47] Wiklund H, Aden AS, Högberg U, Wikman M, Dahlgren L (2000). Somalis giving birth in Sweden: a challenge to culture and gender specific values and behaviours. Midwifery.

[48] McFadden A, Renfrew MJ, Atkin K (2013). Does cultural context make a difference to women’s experiences of maternity care? A qualitative study comparing the perspectives of breast-feeding women of Bangladeshi origin and health practitioners. Health Expect.

[49] Qureshi R, Pacquiao DF (2013). Ethnographic study of experiences of Pakistani women immigrants with pregnancy, birthing, and postpartum care in the united states and Pakistan. J Transcult Nurs.

[50] Rice PL (2000). Rooming-in and cultural practices: choice or constraint?. J Reprod Infant Psychol.

[51] Wilson D, Barton P (2012). Indigenous hospital experiences: a New Zealand case study. J Clin Nurs.

[52] Chalmers B, Omer-Hashi K (2002). What Somali women say about giving birth in Canada. J Reprod Infant Psychol.

[53] Harle MT, Dela RF, Veloso G, Rock J, Faulkner J, Cohen MZ (2007). The Experience of Filipino American Patients With Cancer. Oncol Nurs Forum.

[54] Pasco AC, Morse JM, Olson JK (2004). The cross-cultural relationships between nurses and Filipino Canadian patients. J Nurs Scholarsh.

[55] Ameresekere M, Borg R, Frederick J, Vragovic O, Saia K, Raj A (2011). Somali immigrant women’s perceptions of Cesarean delivery and patient–provider communication surrounding female circumcision and childbirth in the USA. Int J Gynecol Obstet.

[56] Murray L, Windsor C, Parker E, Tewfik O (2010). The experiences of African women giving birth in Brisbane. Australia. Health Care Women Int..

[57] Suurmond J, Uiters E, De Bruijne MC, Stronks K, Essink-Bot M-L (2011). Negative health care experiences of immigrant patients: a qualitative study. BMC Health Serv Res.

[58] Binder P, Johnsdotter S, Essén B (2012). Conceptualising the prevention of adverse obstetric outcomes among immigrants using the ‘three delays’ framework in a high-income context. Soc Sci Med.

[59] Arnaert A, Schaack G (2006). Cultural awareness of Inuit patients’ experiences with emergency nursing care. Accid Emerg Nurs.

[60] Maputle MS, Jali MN (2006). Dealing with diversity: incorporating cultural sensitivity into midwifery practice in the tertiary hospital of Capricorn district, Limpopo province. Curationis.

[61] Berggren V, Bergstrom S, Edberg AK (2006). Being different and vulnerable: experiences of immigrant African women who have been circumcised and sought maternity care in Sweden. J Transcult Nurs.

[62] Jonkers M, Richters A, Zwart J, Öry F, van Roosmalen J (2011). Severe maternal morbidity among immigrant women in the Netherlands: patients’ perspectives. Reprod Health Matter.

[63] Reitmanova S, Gustafson DL (2008). “They can’t understand it”: maternity health and care needs of immigrant Muslim women in St. John’s, Newfoundland. MaternChild Health J.

[64] Wilson DW (2010). Culturally competent psychiatric nursing care. J Psychiatr Ment Hlt.

[65] Binder P, Borné Y, Johnsdotter S, Essén B (2012). Shared language is essential: communication in a multiethnic obstetric care setting. J Health Commun.

[66] Eckhardt R, Mott S, Andrew S (2006). Culture and communication: identifying and overcoming the barriers in caring for non-english-speaking German patients. Divers Health Soc Care.

[67] Garrett PW, Dickson HG, Roberto-Forero, Whelan AK, Lis-Young (2008). What do non-english-speaking patients value in acute care? cultural competency from the patient’s perspective: a qualitative study. Ethnic Health.

[68] Johnson SK (2002). Hmong health beliefs and experiences in the western health care system. J Transcult Nurs.

[69] Lundberg PC, Gerezgiher A (2008). Experiences from pregnancy and childbirth related to female genital mutilation among Eritrean immigrant women in Sweden. Midwifery.

[70] Niner S, Kokanovic R, Cuthbert D (2013). Displaced mothers: birth and resettlement. Gratitude and Complaint. Med Anthropol..

[71] Vangen S, Johansen REB, Sundby J, Træen B, Stray-Pedersen B (2004). Qualitative study of perinatal care experiences among Somali women and local health care professionals in Norway. Eur J Obstet Gyn RB.

[72] Vydelingum V (2000). South Asian patients’ lived experience of acute care in an english hospital: a phenomenological study. J Adv Nurs.

[73] Watson J, Hodson K, Johnson R, Kemp K (2002). The maternity experiences of indigenous women admitted to an acute care setting. Aust J Rural Health.

[74] Hanrahan MC (2002). Identifying the needs of Innu and Inuit patients in urban health settings in Newfoundland and Labrador. Can J Public Health.

[75] Essén B, Binder P, Johnsdotter S (2011). An anthropological analysis of the perspectives of Somali women in the west and their obstetric care providers on caesarean birth. J Psychosom Obst Gyn.

[76] Hill N, Hunt E, Hyrkas K (2012). Somali immigrant women’s health care experiences and beliefs regarding pregnancy and birth in the united states. J Transcult Nurs.

[77] Garrett PW, Dickson HG, Lis-Young, Whelan AK (2008). “The happy migrant effect”: perceptions of negative experiences of healthcare by patients with little or no english: a qualitative study across seven language groups. Qual SafHealth Care.

[78] Lim JW, Baik OM, Ashing-Giwa KT (2012). Cultural health beliefs and health behaviors in Asian american breast cancer survivors: a mixed-methods approach. Oncol Nurs Forum.

[79] Lee T-Y, Landy C, Wahoush O, Khanlou N, Liu Y-C, Li C-C (2014). A descriptive phenomenology study of newcomers’ experience of maternity care services: Chinese women’s perspectives. BMC Health Serv Res.

[80] Hoang HT, Le G, Kilpatrick S (2009). Having a baby in the new land: a qualitative exploration of the experiences of Asian migrants in rural Tasmania, Australia. Rural Remote Health.

[81] Liamputtong P, Watson LF (2006). The meanings and experiences of Cesarean birth among Cambodian, Lao and Vietnamese immigrant women in Australia. Women Health.

[82] Killoran M, Moyer A (2006). Surgical treatment preferences in Chinese-American women with early-stage breast cancer. Psycho-Oncol.

[83] Leininger MM (1988). Leininger’s theory of nursing: cultural care diversity and universality. Nurs Sci Quart.

[84] Campinha-Bacote J (2002). The process of cultural competence in the delivery of healthcare services: A model of care. J Transcult Nurs.

[85] Kleinman A, Benson P (2006). Anthropology in the clinic: the problem of cultural competency and How to Fix It. PLoS Med.

[86] Gastmans C, Dierckx De Casterle B, Schotsmans P (1998). Nursing considered as moral practice: a philosophical-ethical interpretation of nursing. Kennedy Inst Ethics J.

[87] Gastmans C (2013). Dignity-enhancing nursing care: a foundational ethical framework. Nurs Ethics.

[88] Schotsmans P (1999). Personalism in Medical Ethics. Ethical Perspect.

[89] Davidhizar R, Giger J, Hannenpluf L (2006). Your continuing education topic 3 2005: using the giger-davidhizar transcultural assessment model (GDTAM) in providing patient care. J Pract Nurs.

[90] Dierckx de Casterle B (2015). Realising skilled companionship in nursing: a utopian idea or difficult challenge?. J Clin Nurs.

[91] Davidhizar R, Giger J (2002). The giger and davidhizar transcultural assessment model. J Transcult Nurs.

[92] Giger J, Davidhizar R, Fordham P (2006). Multi-cultural and multi-ethnic considerations and advanced directives: developing cultural competency. J Cult Divers.

[93] Warren N (2012). Involving patient and family advisors in the patient and family-centered care model. Med Surg Nurs.

[94] Woodward J, Rice E (2015). Case management. Nurs Clin N Am.

[95] Tichen A (2000). Professional craft knowledge in patient-centered nursing and facilitation of its development.

[96] Claessens P, Dierckx De Casterle B (2003). Skilled companionship: verpleegkundige zorg vanuit een zorgethisch perspectief. Tijdschrift voor gezondheidszorg & ethiek.

[97] Small R, Roth C, Raval M, Shafiei T, Korfker D, Heaman M, Mccourt C, Gagnon A (2014). Immigrant and non-immigrant women’s experiences of maternity care: a systematic and comparative review of studies in five countries. BMC Pregnancy Childbirth.

