# Supplementary material for: “How do ethnic minority patients experience the intercultural care encounter in hospitals? a systematic review of qualitative research”
Source: BMC Med Ethics. 2017 Jan 19;18:2. doi: 10.1186/s12910-016-0163-8 (PMC5244561; doi:10.1186/s12910-016-0163-8)
Supplement: Additional file 1: — Full Search String. String adjustments for each database with the results (PDF 91 kb) [file 12910_2016_163_MOESM1_ESM.pdf]

Additional file 1: Full Search String

Database	Date	String	Search	Results
Pubmed	03/03/2015	(multicultural OR intercultural OR cross-cultural OR transcultural) AND (multicultural OR intercultural OR cross-cultural OR transcultural OR ethnic groups OR migration OR immigrants OR minority) AND (views OR experiences OR attitudes OR perspectives OR qualitative research OR opinions) AND (hospitalization OR hospital OR institutionalization OR health care OR healthcare OR nursing)	Advanced	n = 5397
Cinahl	03/03/2015	(multicultural OR intercultural OR cross-cultural OR transcultural) AND (multicultural OR intercultural OR cross-cultural OR transcultural OR ethnic groups OR migration OR immigrants OR minority) AND (views OR experiences OR attitudes OR perspectives OR qualitative research OR opinions) AND (hospitalization OR hospital OR institutionalization OR health care OR healthcare OR nursing)	Advanced<br>*(no "suggest subject terms")*	n = 1188
Medline	03/03/2015	(multicultural OR intercultural OR cross-cultural OR transcultural) AND (multicultural OR intercultural OR cross-cultural OR transcultural OR ethnic groups OR migration OR immigrants OR minority) AND (views OR experiences OR attitudes OR perspectives OR qualitative research OR opinions) AND (hospitalization OR hospital OR institutionalization OR health care OR healthcare OR nursing)	Multi-field	n = 2733
Web of Science	03/03/2015	(multicultural OR intercultural OR cross-cultural OR transcultural) AND (multicultural OR intercultural OR cross-cultural OR transcultural OR ethnic groups OR migration OR immigrants OR minority) AND (views OR experiences OR attitudes OR perspectives OR qualitative research OR opinions) AND (hospitalization OR hospital OR institutionalization OR health care OR healthcare OR nursing)	Basic search	n = 1485
Embase	03/03/2015	(multicultural OR intercultural OR **cross cultural** OR transcultural) AND (multicultural OR intercultural OR **cross cultural** OR transcultural OR ethnic groups OR migration OR immigrants OR minority) AND (views OR experiences OR attitudes OR perspectives OR qualitative research OR opinions) AND (hospitalization OR hospital OR institutionalization OR health care OR healthcare OR nursing)	Quick search<br>*(no "search as broadly as possible")*	n = 2043
