# Supplementary material for: “How do ethnic minority patients experience the intercultural care encounter in hospitals? a systematic review of qualitative research”
Source: BMC Med Ethics. 2017 Jan 19;18:2. doi: 10.1186/s12910-016-0163-8 (PMC5244561; doi:10.1186/s12910-016-0163-8)
Supplement: Additional file 2: — Summary of Characteristics of the Included Articles. A summary of the characteristics of all the included articles (PDF 555 kb) [file 12910_2016_163_MOESM2_ESM.pdf]

Additional file 2: Summary of characteristics of the included articles

	Author(s) / Year	Care setting/ Country	Aim(s) of the study	Method	Sample & ethnicity patient	Theoretical framework & data analysis	Ethical considerations
1	**Amereskere et al.** (2011)	Maternity care, US	To explore Somali women's' perceptions of caesarean delivery and patient-provider communication surrounding female circumcision and childbirth	Interview study	23 Somali women	Iterative approach of analysis	Approval by the institutional Review Board of Boston University Medical Campus
2	**Arnaert and Schaack** (2006)	Emergency care, Canada	To explore Inuit patients' experiences with emergency nursing in an urban Health Centre, to inform on the best holistic nursing practices to meet culturally specific needs	Interview study	4 Inuit patients	Comparative method by Strauss and Corbin	Approval by the institution's Ethical Review Board and the Nunavik Regional Board of Health and Social services
3	**Baker and Daigle** (2000)	Hospital care, Canada	To explore the hospitalization experiences of members of the Mi'kmaq, First Nation Community to understand cultural sensitivity from their perspectives	Interview and thick descriptions study	10 Mi'kmaq patients	Interpretive Interactionistic approach by Denzin	Efforts to deal with ethical considerations:<br>- informed consent<br>- anonymity<br>-confidentiality
4	**Berggren, Bergstrom and Edberg** (2006)	Maternity care, Sweden	To explore the experience of Female genital cutting and the experiences of encounters with Swedish maternal health care among women from Somalia, Eritrea, and Sudan living in Sweden	Interview study	22 migrant women (Somalia, Sudan and Eritrea)	Latent content analysis by Berg and Weber	Approval by Ethics Committee of Karolinska Institute
5	**Binder et al. *** (2012)	Maternity care, England	To explore the multi-ethnic care setting and the roles that ethnicity and language play during the care encounter between immigrant women and their western obstetric care providers	Interview and focus group study	39 Somali women *(reported)*<br>1 Ghanaian women *(reported)*<br>10 white British women *(not reported)*<br>62 obstetric caregivers *(not reported)*	Analysis according to naturalistic inquiry	Approval by the Research Ethics Committee of NHS, United Kingdom

	Author(s) / Year	Care setting/ Country	Aim(s) of the study	Method	Sample & ethnicity patient	Theoretical framework & data analysis	Ethical considerations
6	**Binder, Johnsdotter and Essén** (2012)	Maternity care, England	To explore the influence of pre-migration socio-cultural factors on post-migration maternal care seeking and barrier-causing delays to women's utilisation of facility care and to understand the reasons behind barriers between immigrant women and maternal care providers.	Interview and focus group study	54 migrant women (sub-Saharan regions, Africa)<br>62 maternal caregivers *(not reported)*	Constructivist Hermeneutic Naturalistic approach	Approval by the Research Ethics Committee of NHS, United Kingdom
7	**Chalmers and Omer-Hashi** (2002)	Maternity care, Canada	To explore the experiences of Somali women who had recently given birth to a baby in Canada and to gain information about these women's perceptions of their recent care during pregnancy and birth	***Mixed-method study***<br>- qualitative interviews *(reported)*<br>- quantitative closed-ended interviews *(not reported)* *(part of larger mixed method study*	432 Somali women	Descriptive content analysis	Approval by the Ethics Committee of Women's College Hospital in Toronto
8	**Cheragi, Manookian and Nasrabadi** (2014)	Hospital care, Iran	To identify common experiences of Iranian Muslim and Armenian Christian patients regarding dignified care at the bedside	Interview study (qualitative part of larger study)	5 Iranian Armenians *(reported)*<br>5 Iranian Muslims *(not reported)*	Inductive content analysis	Approval by the Ethics Committee of Tehran University of Medical Sciences
9	**Cheung **** (2002a)	Maternity care, Scotland	To provide insights as to how women's childbearing experiences might be improved	***Interview, participant observational and literature review stud***<br>- semi-structured interviews (patients, reported)<br>- unstructured interviews (health workers, relatives and friends, not reported)<br>- participant observations (patients, reported)<br>- literature review and case comparison (not reported)	10 Chinese women *(reported)*<br>10 Scottish women *(not reported)*<br>45 health workers; relatives and friends *(not reported)*	Comparative analysis	Approval by the Ethics Committee of the local Health Board

	Author(s) / Year	Care setting/ Country	Aim(s) of the study	Method	Sample & ethnicity patient	Theoretical framework & data analysis	Ethical considerations
10	**Cheung **** (2002b)	Maternity care, Scotland	To analyse the meanings that women gave to their childbearing experiences in order to provide some useful insights as to how their experiences might be improved	***Interview, participant observational and literature review study***<br>- semi-structured interviews *(patients, reported)*<br>- unstructured interviews *(health workers, relatives and friends, not reported)*<br>- participant observations *(patients, reported)*<br>- literature review and case comparison *(not reported)*	10 Chinese women *(reported*<br>10 Scottish women *(not reported)*<br>45 health workers; relatives and friends *(not reported)*	Anthropological contemporary fieldwork approach<br>1.) Textual and content analysis<br>2.) Contextual, comparative analysis	Approval by the Ethics Committee of the local Health Board
11	**Cortis ***** (2000a)	Hospital care, UK	To explore the Pakistani immigrants' concept of care and their experiences of care delivered in hospital in UK	Interview study	20 male Pakistani<br>18 female Pakistani	Framework of Leininger's Theory of Culture Care Diversity and Universality & Leininger's four phase of ethnonursing data analysis	/
12	**Cortis** (2000b)	Hospital care, UK	To explore the lived experiences of Pakistani communities that received nursing care in a hospital setting	Interview and focus group study	30 male Pakistani Muslim (Urdu-speaking)<br>25 female Pakistani Muslim (Urdu-speaking)	Grounded theory approach	/
13	**Cortis and Kendrick ***** (2003)	Hospital care, UK	To explore the expectations and perceived experiences of nursing care among members of the Pakistani community in UK	Interview study	20 male Pakistani<br>18 female Pakistani	Theoretical framework of Leininger's Theory of Culture Care Diversity and Universality & Leininger's four phase of ethnonursing data analysis	/

	Author(s) / Year	Care setting/ Country	Aim(s) of the study	Method	Sample & ethnicity patient	Theoretical framework & data analysis	Ethical considerations
14	**Eckhardt and Mott and Andrew** (2006)	Hospital care, Australia	To explore the experiences of six older German- born women who had undergo planned admission to hospital in Australia	Interview study	6 German	Descriptive phenomenology approach & content or theme analysis	Approval by institutional Ethics Committee
15	**Essén et al.** (2000)	Maternity care, Sweden	To explore attitudes, strategies and habits of Somalian immigrant women related to pregnancy and childbirth in order to understand how cultural factors might affect perinatal outcome	Interview study	15 Somali women	Medical-anthropological approach & systematic text analysis	Approval by the Ethical Research Committee of Lund University
16	**Essén, Binder and Johnsdotter *** (2011)	Maternity care, England	To explore the relationship between Somali women and their western obstetric care providers. The attitudes, perceptions, beliefs and experiences will be explored in relation to caesarean birth	Interview and focus group study	39 Somali women *(reported)*<br>62 obstetric care providers *(not reported)*	Anthropological naturalistic inquiry approach	Approval by the Riverside Research Ethics Committee of London
17	**Fenwick and Stevens** (2004)	Surgical care, Australia	To explore the postoperative pain experiences of Central Australian Aboriginal women	***Interview, focus group and participant observational study***<br>- interview and participant observation *(Aboriginal women, reported)*<br>- interview and participant observation *(nurses, not reported)*<br>- focus group *(health workers, not reported)*	5 Aboriginal female patients *(reported)*<br>8 non-Aboriginal female nurses *(not reported)*<br>4 Aboriginal female health workers *(not reported)*	Grounded theory approach & constant comparative analysis	Approval by Southern Cross University Research Ethics Committee and the Aboriginal Ethics subcommittee attached to the regional hospital
18	**Garrett et al.****** (2008a)	Hospital care, Australia	To explore the experiences of patients with little or no English who either attended the Emergency Department of were admitted to the wards of a tertiary referral hospital	Language-specific focus group study *(Part of larger study)*	49 patients *(reported)* (Vietnamese, Italian, Arabic, Spanish, Croatian, Chinese and Serbian speaking)<br>9 carers of these patients *(not reported)*	Grounded theory approach	Approval by the Human Research Ethics Committee of the Sydney South West Area Health Service (Western Zone)

	Author(s) / Year	Care setting/ Country	Aim(s) of the study	Method	Sample & ethnicity patient	Theoretical framework & data analysis	Ethical considerations
19	**Garrett et al.****** (2008b)	Hospital care, Australia	To explore conceptions of cultural competence in the experiences of non-English speaking acute care patients	Language-specific focus group study *(Part of larger study)*	49 acute hospital patients *(reported)* (Vietnamese, Italian, Arabic, Spanish, Croatian, Chinese and Serbian speaking)<br>10 carers from non-English-speaking background *(not reported)*	Grounded theory approach & constructivist perspective	Approval by the South Western Sydney Area Health Service Ethics Committee
20	**Grewal, Bhagat and Balneaves** (2008)	Maternity care, Canada	To explore new immigrant Punjabi women's perinatal experiences and the ways that traditional beliefs and practices are incorporated into the Canadian health care context	***Interview and focus group study***<br>- Interview study *(mothers, reported)*<br>- Focus groups study *(experts, not reported)*	15 Punjab mothers *(reported)*<br>5 health professionals and community leaders *(not reported)*	Naturalistic, descriptive approach & line-by-line analysis	Approval by Institutional Ethical Committee
21	**Hanrahan** (2002)	Hospital care, Canada	To explore Innu and Inuit people's experiences of health services in the urban centres of St. John's and Goose Bay	***Interview and focus group study***<br>- interviews *(patients, reported)*<br>- interviews *(caregivers, not reported)*	25 Innu patients<br>118 Inuit patients<br>8 health care practitioners *(not reported)*	Content analysis	Approval by the Human Investigation committee of the Health Care Corporation of St. John's and Faculty of Medicine
22	**Harle et al.** (2007)	Cancer care, US	To explore the experiences of Filipino patients with cancer and to inform nurses in delivering culturally appropriate care	Focus group study	18 female Filipino patients<br>5 male Filipino patients	Hermeneutic phenomenological approach & phenomenological analysis	Approval by the Institutional Review Board of a major comprehensive cancer center and its affiliated university

	Author(s) / Year	Care setting/ Country	Aim(s) of the study	Method	Sample & ethnicity patient	Theoretical framework & data analysis	Ethical considerations
23	**Herrel et al.** (2004)	Maternity care, US	To explore how Somali women have experienced pregnancy and Childbirth in Minnesota	Focus group study	14 Somali women	/	Approval by the Park Nicollet Institute Institutional Review Board
24	**Higginbottom et al.** (2013)	Maternity care, Canada	To explore the maternity experiences of women of Sudanese origin in an urban Canadian city	Focus group study *(Part of larger mixed-method study)*	12 Sudanese women	Focused ethnography approach & ethnographic analysis by Roper and Shapira	Approval by the institutional review board of the University of Alberta
25	**Higginbottom et al.** (2015)	Maternity care, Canada	To identify the nature of communication difficulties and to gain insight into the issues related to maternity care access and care provision from the perspectives of immigrant women	Interview study (*Part of larger mixed-method study)*	12 women *(reported) (Africa, Pakistan, Congolese-Canadian, Philippines, Eritrea)*, 12 health care providers *(not reported* 6 social service providers *(not reported)*	Focused ethnography approach & ethnographic analysis by Roper and Shapira	Approval by the Ethics Board of University of Alberta
26	**Hill, Hunt and Hyrkas** (2012)	Maternity care, US	To explore Somali immigrant women's health care experiences and beliefs regarding pregnancy and birth in the US	Focus group study	18 Somali women	Thematic content analysis	Approval by the institutional Review Board of the hospital
27	**Hoang and Kilpatrick** (2009)	Maternity care, Australia	To explore Asian migrant women's child-birth experiences in a rural Australian context	Interview study	11 Asian women	Grounded theory approach by Strauss and Corbin	Approval by the Tasmanian Social Sciences Human Research Ethics Network

	Author(s) / Year	Care setting/ Country	Aim(s) of the study	Method	Sample & ethnicity patient	Theoretical framework & data analysis	Ethical considerations
28	**Johnson** (2002)	Primary care clinic, US	To explore Hmong perspectives and beliefs that influence the Hmong experience in Western medical situations	Interview, focus group and participant observational study	11 Hmong women (Laos)<br>8 Hmong men (Laos)	Ethnographic approach	Approval by the Committee on Human Research of San Francisco State University
29	**Jonkers et al.** (2011)	Maternity care, Netherlands	To explore ethnicity-related factors contributing to sub-standard maternity care and the effects on severe maternal morbidity among immigrant women in the Netherlands	Interview study	40 immigrant women *(reported)*<br>10 native Dutch women *(not reported)*	Thematic analysis	Approval by Medical Ethical Committee of Leiden University Medical Center
30	**Killoran and Moyer** (2006)	Oncology care, US	To explore the cultural factors that influence the selection of treatments by Chinese-American patients	Interview, focus group and observational study	69 Chinese-American women *(reported)*<br>14 health professionals *(not reported)*	Ethnographic approach & comparative triangulation analysis	Efforts to deal with ethical considerations:<br>- verbal informed consent
31	**Lee et al.** (2014)	Maternity care, Canada	To explore immigrant Chinese women's experiences in accessing maternity care, the utilization of maternity health services, and the obstacles they perceived in Canada	Interview study	15 Chinese patients	Descriptive phenomenological approach & phenomenological analysis by Colaizzi	Approval by Research Ethics Board of York University's
32	**Liamputtong and Watson** (2006)	Maternity care, Australia	To explore the experience of caesarean birth among 67 Cambodian, Lao, and Vietnamese women living in Australia	Interview study *(part of larger ethnographic study)*	67 women, *(21 from Laos, 21 Vietnam, 25 Cambodia)*	Thematic analysis	Approval by La Trobe University Human Ethics Committee.

	Author(s) / Year	Care setting/ Country	Aim(s) of the study	Method	Sample & ethnicity patient	Theoretical framework & data analysis	Ethical considerations
33	**Lim, Baik and Ashing-Giwa** (2012)	Hospital care, US	To explore the relationships between cultural health beliefs, acculturation, treatment-related decisions, doctor-patient relationship and health behaviours among Asian American breast cancer survivors and the contextual meaning of those relationships among Korean American breast cancer survivors and Asian American breast cancer survivors	***Mixed-method study***<br>- qualitative focus groups study *(reported)*<br>- quantitative survey study *(not reported)*	11 Korean American breast cancer survivors *(reported)*	Descriptive, exploratory approach	Approval by the institutional Review Board of City of Hope National Medical Center
34	**Lundberg and Gerezgiher** (2006)	Maternity care, Sweden	To explore Eritrean immigrant women's experiences of female genital mutilation during pregnancy, childbirth and the postpartum period	Interview study	15 Eritrean women	Ethnographic approach & thematic analysis	Efforts to deal ethical considerations:<br>- written and oral presentation<br>- written informed consent<br>- anonymity<br>- confidentiality<br>- coding of informant<br>-destroying tape recordings
35	**Maputle and Jali** (2006)	Maternity care, South Africa	To explore the current and patterned care life ways of mothers from diverse cultural groups that deliver their babies at the hospital in Limpopo Province	Interview study with semi-structured and unstructured conversations	24 women from diverse ethnic groups (N.Sotho, Tsonga, Venda)	Ethnographic approach, analysis according to Mile's and Huberman Theoretical framework of Leininger's Sunrise Model theory	Efforts to deal with ethical considerations:<br>- Informed consent

	Author(s) / Year	Care setting/ Country	Aim(s) of the study	Method	Sample & ethnicity patient	Theoretical framework & data analysis	Ethical considerations
36	**McFadden, Renfrew and Atkin** (2013)	Maternity care, England	To explore the extent to which cultural context makes a difference to experiences of breast-feeding support for women of Bangladeshi origin and to consider the implications for the provision of culturally appropriate care	Interview and focus group study<br>- interviews *(women, reported)*<br>- focus group *(health practitioners, not reported)*	23 women of Bangladeshi origin<br>4 health service managers *(not reported)*	/	Approval by the Research Ethics Committee of North West NHS
37	**Missal, Clark and Kovaleva** (2015)	Maternity care, US	To explore Somali immigrant new mother's experience of childbirth in Minnesota	Interview study	12 Somali women	Framework of Leininger's Theory of Culture Care Diversity and Universality & Leininger's and McFarland's four phase of ethnonursing data analysis	Approval by the institutional Review Board of the university
38	**Murray et al.** (2009)	Maternity care, Australia	To explore the birth experiences of African refugee women in Australia	Interview study	10 African refugees	Husserlian phenomenological framework & analysis according to Amedo Giorgi	Approval by Technology Human Research Ethics Committee of Queensland University
39	**Niner, Kokanovic and Cuthbert** (2013)	Obstetric care, Australia	To explore the effects of inequality consequent on displacement through a focus on the birthing experiences of forced migrant women in Australian hospitals	Interview study	15 Karen women *(Burma-Myanmar)*	Ethnographical approach	/
40	**Pasco, Morse and Olson** (2004)	Hospital care, Canada	To describe culturally embedded values that implicitly guide Filipino Canadian patient's interactions with Canadian nurses and are integral to nurse-patient relationships	Interview study *(ethnographic interviews, field notes and diary)*	23 Filipino patients	Focused ethnographical approach	Approval by appropriate University and Ethical Agency
41	**Qureshi and Pacquiao** (2013)	Maternity care, US	To explore the comparative birthing experiences of Pakistani immigrant women in Pakistan and the united states	Interview study	26 Pakistani women	Ethnographical approach by Berry's framework	Efforts to deal with ethical considerations:<br>-Informed consent

	Author(s) / Year	Care setting/ Country	Aim(s) of the study	Method	Sample & ethnicity patient	Theoretical framework & data analysis	Ethical considerations
42	**Reitmanova and Gustafson** (2008)	Maternity care, Canada	To explore the maternity health care needs and barriers to accessing maternal health services from the perspective of immigrant Muslim women living in Canada	Interview study	6 Muslim women	Thematic analysis	Approval by Research Ethics Committee of University
43	**Rice** (2000)	Maternity care, Australia	To explore the policy of rooming-in within maternity hospitals in Australia and to argue for a more flexible policy when providing care to women from Asian backgrounds	Interview study	27 Hmong women<br>8 Vietnamese women<br>8 Cambodian women	Thematic analysis	Efforts to deal with ethical considerations:<br>-Informed consent
44	**Suurmond et al.** (2011)	Hospital care, Netherland	To explore negative events in hospital care as perceived by immigrant patients	Interview study *(individual and group interviews)*	22 respondents *(Turkish, Chinese, Italian, Dominican, Chilean, Portuguese and Surinamese origin)*	Framework analysis	Efforts to deal with ethical considerations:<br>-anonymity respondents<br>-informed consent
45	**Vangen et al.** (2003)	Maternity care, Norway	To explore how perinatal care practice may influence labour outcomes among circumcised women	Interview study	23 Somali women *(reported)*<br>36 Norwegian health care professionals *(not reported)*	Systematical analysis	Approval by Regional Ethical Research Committee
46	**Vyndelingum** (2000)	Acute care, England	To explore the 'lived experiences' of acute hospital care from South Asian patients	Interview study	10 patients *(Hindus, Muslims, Sikhs) (reported)*<br>6 carers *(not reported)*	Heideggerian hermeneutics phenomenological approach	Approval by local Ethics Committee
47	**Watson et al.** (2002)	Acute maternity care, Australia	To explore maternity experiences of indigenous women admitted to an acute care setting prior to or after the birth of their baby	Interview study *(informal "chat style") (part of larger three-stage project)*	12 indigenous women *(Aboriginal and Torres Strait Islander)*	Descriptive approach	Approval prior to commencement of three-stage project

	Author(s) / Year	Care setting/ Country	Aim(s) of the study	Method	Sample & ethnicity patient	Theoretical framework & data analysis	Ethical considerations
48	**Wikberg, Eriksson and Bondas** (2012)	Maternity care, Finland	To explore perceptions and experiences of caring of immigrant new mothers from an intercultural perspective in maternity care in Finland	Interview and observational study	17 women from 12 countries *(Australia, Bosnia, Burma, Colombia, Estonia, Hungary, India, Iraq, Russia, Thailand, Uganda, Vietnam)*	Focused ethnography and Eriksson's theory of caritative caring approach & analysis according to Roper & Shapira	Approval by Ethical Committee of the hospital and recommendations of the Finnish National Advisory Board on Research Ethics were followed
49	**Wiklund et al.** (2000)	Maternity care, Sweden	To explore childbirth experiences of Somali women in Sweden and to study the meeting between Somali women giving birth in Sweden and Swedish antenatal and delivery care.	Interview study	9 Somali women *(reported)*<br>7 Somali men *(not reported)*	Grounded theory approach	Approval by the Ethical Research Committee of Umea University
50	**Wilson** (2010)	Mental health care clinic, US	To explore the perceptions of clients with mental illness regarding the overall effectiveness of psychiatric nursing care in meeting their cultural needs	***Mixed-method study***<br>- qualitative interview study *(reported)*<br>- quantitative self-administered nurse questionnaire *(not reported)*	16 African American patients *(reported)*<br>65 Registered Nurses *(not reported)*	Descriptive approach & thematic content analysis according to Kvale and Patton	Efforts to deal with ethical considerations:<br>- Written consent (University's Institutional Review Board policies and guidelines)
51	**Wilson and Barton** (2012)	Hospital care, New Zealand	To explore Maori experiences of hospitalisation in surgical or medical settings and how these might influence length of stay	***Mixed-method study***<br>qualitative interview study *(reported)*<br>- quantitative retrospective interrupted time series study *(not reported)*<br>- structured review of the literature *(not reported)*<br>- medical-surgical discharge data *(not reported)*	11 Maori (indigenous) patients	Maori centred approach with case study methodology & thematic analysis	Approval by the University Ethics Committee

* Essén, Binder & Johnsdotter (2011) and Binder (2012), ** Cheung (2002a) and (2002b), *** Cortis (2000a) and Cortis and Kendrick (2003) , **** Garret (2008a) and Garret (2008b) are respectively estimated as part of the same study
