# Supplementary material for: “How do ethnic minority patients experience the intercultural care encounter in hospitals? a systematic review of qualitative research”
Source: BMC Med Ethics. 2017 Jan 19;18:2. doi: 10.1186/s12910-016-0163-8 (PMC5244561; doi:10.1186/s12910-016-0163-8)
Supplement: Additional file 3: — Sensitivity Analysis. The results of the sensitivity analysis carried out for each included article (PDF 339 kb) [file 12910_2016_163_MOESM3_ESM.pdf]

Additional file 3: Sensitivity Analysis

		Clear description of…							Sensitivity analysis		
**Nr**	**Qualitative studies**	aim/research question	background literature	study design and procedure	sampling method, incl./excl. criteria	data collection and analysis	ethical considerations	results	**Rigor** (result rapportage)	**Relevance**	**Relative contribution** (medium, high, low)*
1.	Ameresekere et al. (2011)	+	+	+/-	+	+	+	+/-	+	+/-	Medium (Low)
2.	Arnaert & Schaack (2006)	+	+	+/-	+	+	+	+	+	+	High
3.	Baker & Daigle (2000)	+	+	+	+	+	+	+	+	+	High
4.	Berggren et al. (2006)	+	+	+	+	+	+	+	+	+	High
5.	Binder et al. (2012a)	+	+	+/-	+	+	+	+	+	+	High
6.	Binder et al. (2012b)	+	+	+	+	+	+	+	+	+/-	Medium (High)
7.	Chalmers & Omer-Hashi (2002)	+	+	+/-	+	+	+	+	+	+	High
8.	Cheraghi et al. (2014)	+	+	+/-	+	+	+	+	+	+	High
9.	Cheung (2002a)	+	+	+	+/-	+	+	+	+	+	High
10.	Cheung (2002b)	+	+	+	+/-	+	+	+	+	+	High
11.	Cortis (2000a)	+	+	+	+	+	-	+	+	+	High
12.	Cortis (2000b)	+	+/-	+	+	+	-	+	+	+	High
13.	Cortis & Kendrick (2003)	+	+	+	+	+	-	+	+	+	High
14.	Eckhardt et al. (2006)	+	+	+	+	+	+	+	+	+	High
15.	Essén et al. (2011)	+	+	+	+	+	+	+	+	+/-	Medium (Low)
16.	Essén et al. (2000)	+	+	+	+	+	+	+	+	+/-	Medium (Low)

17.	Fenwick & Stevens (2004)	+	+	+	-	+	+	+/-	+	+	High
18.	Garrett et al.(2008a)	+	-	+	+/-	+/-	+	-	-	+	Medium (High)
19.	Garret et al. (2008b)	+	+	+	+/-	+	+	+	+	+	High
20.	Grewal et al. (2008)	+	+	+	+	+	+	+	+	+/-	Medium (High)
21.	Hanrahan (2002)	+	-	+/-	+/-	+	+	+/-	-	-	Low
22.	Harle et al. (2007)	+	+	+	+	+	+	+	+	+/-	Medium (High)
23.	Herrel et al. (2004)	+	+/-	+/-	+/-	+/-	+	+	+/-	+	Medium (High)
24.	Higginbottom et al. (2013)	+	+	+	+	+	+	+	+	+/-	Medium (High)
25.	Higginbottom et al. (2015)	+	+	+	+	+	+	+	+	+	High
26.	Hill et al. (2012)	+	+	+/-	+	+	+	+/-	+	+/-	Medium (High)
27.	Hoang et al. (2009)	+	+	+	+	+	+	+	+	+	High
28.	Johnson (2002)	+	+	+	+/-	-	+	+	+	+	High
29.	Jonkers et al. (2011)	+	-	+/-	+/-	+	+	+	+/-	+	Medium (High)
30.	Killoran & Moyer (2006)	+	+	+/-	+	+	+/-	+	+	+/-	Medium (Low)
31.	Lee et al. (2014)	+	+	+	+	+	+	+	+	+	High
32.	Liamputtong & Watson (2006)	+	+	+	+	+	+	+	+	+	High
33.	Lim et al.(2012)	+	+	+	+	+	+	+	+	+	High
34.	Lundberg & Gerezgiher (2008)	+	+	+	+	+	+	+	+	+/-	Medium (High)
35.	Maputle & Jali (2006)	+	+/-	+	+/-	+/-	+/-	-	-	-	Low
36.	McFadden et al. (2013)	+	+	+/-	+/-	+	+	+	+	-	Medium (Low)

37.	Missal et al. (2015)	+	+	+	+	+	+	+	+	+	High
38.	Murray et al. (2010)	+	+	+	+	+	+	+	+	+	High
39.	Niner et al. (2013)	+	+	+/-	+/-	+/-	-	+	-	+	Medium (High)
40.	Pasco et al. (2004)	+	+	+	+/-	+/-	+	+	+	+	High
41.	Qureshi & Pacquiao (2013)	+	+	+	+	+/-	+/-	+	+	-	Medium (Low)
42.	Reitmanova & Gustafson (2008)	+	+	+/-	+	+	+	+	+	+	High
43.	Rice (2000)	+	+	+/-	+	+	+/-	+	+	+	High
44.	Suurmond et al. (2011)	+	+	+/-	+	+	+	+	+	+	High
45.	Vangen et al. (2013)	+	+	+	+	+	+	+	+	+/-	Medium (High)
46.	Vydelingum (2000)	+	+	+	+	+	+	+	+	+	High
47.	Watson (2002)	+	+	+	+	+/-	+/-	+	+	+	High
48.	Wikberg (2012)	+	+	+	+	+	+	+	+	+	High
49.	Wiklund (2000)	+	+/-	+	+	+	+	+	+	+/-	Medium (High)
50.	Wilson (2012)	+	+	+/-	+/-	+/-	+	+	+/-	+	Medium (High)
51.	Wilson (2010)	+	+	+	+	+	+	+	+	+	High

*in case of a “medium” relative contribution, we nuanced this contribution as being rather “medium /low” or “medium/ high” based on the articles’ relevance and not on their rigor
